# Extracellular Vesicles as Mediators of Neuroinflammation in Intercellular and Inter-Organ Crosstalk

**DOI:** 10.3390/ijms25137041

**Published:** 2024-06-27

**Authors:** Andrea Cabrera-Pastor

**Affiliations:** 1 Departamento de Farmacología, Facultad de Medicina y Odontología, Universitat de València, 46010 Valencia, Spain; acabrera@incliva.es or andrea.cabrera@uv.es; 2 Fundación de Investigación del Hospital Clínico Universitario de Valencia, INCLIVA, 46010 Valencia, Spain

**Keywords:** extracellular vesicles, exosomes, neuroinflammation, neurological disorders, inter-organ crosstalk, glial cells, neuron, CNS barrier

## Abstract

Neuroinflammation, crucial in neurological disorders like Alzheimer’s disease, multiple sclerosis, and hepatic encephalopathy, involves complex immune responses. Extracellular vesicles (EVs) play a pivotal role in intercellular and inter-organ communication, influencing disease progression. EVs serve as key mediators in the immune system, containing molecules capable of activating molecular pathways that exacerbate neuroinflammatory processes in neurological disorders. However, EVs from mesenchymal stem cells show promise in reducing neuroinflammation and cognitive deficits. EVs can cross CNS barriers, and peripheral immune signals can influence brain function via EV-mediated communication, impacting barrier function and neuroinflammatory responses. Understanding EV interactions within the brain and other organs could unveil novel therapeutic targets for neurological disorders.

## 1. Introduction

Neuroinflammation, characterized by immune responses within the central nervous system (CNS), stands as a central player in various neurological disorders, such as Alzheimer’s disease, multiple sclerosis, Parkinson’s disease, and hepatic encephalopathy [1,2]. Elucidating the intricate mechanisms governing neuroinflammation is paramount for the development of effective therapeutic strategies against these debilitating conditions [3,4,5]. In this context, extracellular vesicles (EVs) have emerged as crucial mediators of intercellular communication, facilitating the transfer of bioactive molecules between cells and modulation of immune responses.

Recent research has shed light on the involvement of EVs in neuroinflammation, highlighting their role in propagating inflammatory signals and influencing the progression of diseases [6]. EVs, released by diverse cell types within the nervous system, are documented carriers of specific cargo, including microRNAs, proteins, and lipids, capable of regulating immune responses and neuronal function [7,8,9]. However, the precise mechanisms through which EVs contribute to neuroinflammation and inter-organ crosstalk remain incompletely understood.

Some studies suggest that EVs derived from activated microglia exacerbate inflammation by transferring pro-inflammatory molecules to neighboring cells, while others propose a regulatory role for EVs in dampening excessive immune responses and promoting tissue repair [10,11]. EVs act as vehicles of information, whereby their content and cellular origin dictate their functional role. This underscores the complexity of the specific pathways involved in EV-mediated communication in neuroinflammation.

In pathological states, such as neuroinflammation, neurological and cardiovascular diseases, and metabolic disorders, dysregulated EV signaling pathways contribute to disease progression and complications [12]. Understanding the role of EVs in inter-organ communication during pathological conditions holds significant scientific importance. In fact, there have been a growing number of studies on the intercommunication between the brain and other organs because EVs can function across the blood–brain barrier [13]. Moreover, elucidating the intricate networks of EV communication between organs offers opportunities to develop novel diagnostic biomarkers and therapeutic strategies for mitigating disease progression and improving patient outcomes.

This review intends to summarize the current understanding of the involvement of EVs in neuroinflammation, elucidating some of their mechanisms of action and exploring their therapeutic potential. The goal is to provide a comprehensive overview of the role of EVs in mediating intercellular communication during neuroinflammatory processes. Ultimately, this work aims to contribute to a better understanding of the bidirectional communication networks between the brain and other essential organs (such as the heart, adipose tissue, liver, and intestine), paving the way for innovative therapeutic interventions targeting EVs in neurological disorders.

## 2. Extracellular Vesicles: An Overview

**Figure 1:**
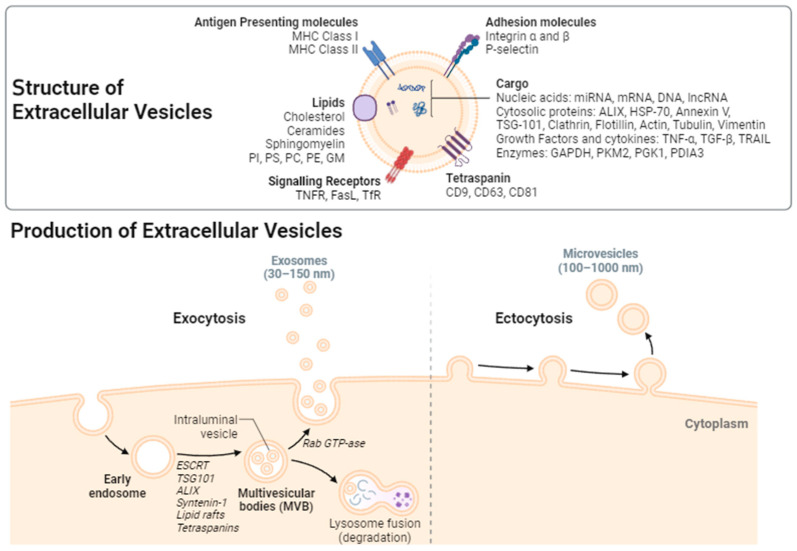
**Biogenesis of Extracellular Vesicles (EVs).** The biogenesis of exosomes involves a tightly regulated process that begins with the formation of early endosomes. These early endosomes mature into multivesicular bodies (MVBs), which contain intraluminal vesicles harboring specific cargo molecules. Some of these MVBs directly fuse with lysosomes and degrade, some are transported to the Golgi for recovery, and some fuse with the cell membrane to release small vesicles outside of the cell and form exosomes. Many molecules play an important role in exosome biogenesis and abscission. First, the endosomal sorting complex required for transport (ESCRT) and other proteins, such as tumor susceptibility gene 101 protein (TSG101) and ALG-2 interacting protein X (ALIX), are involved in cargo sorting into exosomes. In addition, other ESCRT-independent mechanisms, including lipid rafts and tetraspanins CD63 and CD81, are conducive to exosome biogenesis. Finally, the Rab-GTPase family contributes to the intracellular trafficking and fusion of MVBs with the cell membrane to release exosomes. Exosomes are small EVs, typically ranging from 30 to 150 nanometers in diameter. In contrast, microvesicles are formed by the outward budding and shedding of the plasma membrane, resulting in the direct release of vesicles into the extracellular environment. Microvesicles are larger EVs, generally ranging from 100 to 1000 nanometers in size. The cargo of EVs includes a diverse array of bioactive molecules, such as nucleic acids (mRNA, miRNA, DNA, lncRNA), proteins, and lipids. The different surface proteins are transmembrane proteins such as tetraspanins (such as CD9, CD63, CD81), antigen-presenting molecules (MHC I and II), adhesion molecules (such as integrins, P-selectin), and other signaling receptors (such as TNFR, FasL, TfR); proteins in the EV lumen, such as heat shock proteins (HSPs), cytoskeletal proteins (such as actin, tubulin, vimentin), ESCRT components (such as Alix, TSG-101), membrane transport and fusion proteins (such as GTPases, Annexin, Flotillin, Clathrin), growth factors and cytokines (such as TNF-α, TGF-β, TRAIL), and metabolic enzymes (such as GAPDH, PKM2, PGK1, PDIA3). EVs also comprise multiple lipids, such as cholesterol, ceramides, sphingomyelin, phosphatidylinostol (PI), phosphatidylserine (PS), phosphatidylcholine (PC), phosphatidylethanolamine (PE), and gangliosides (GM). Importantly, the composition of EV cargo is influenced by the originating cell type and its physiological state. TNF-α = tumor necrosis alpha, TGF-β = transforming growth factor beta, TRAIL = TNF-related apoptosis-inducing ligand, messenger RNA (mRNA), microRNA (miRNA), lncRNA = long non-coding RNAs, GAPDH = Glyceraldehyde 3-phosphate dehydrogenase, PKM2 = Pyruvate kinase isozyme M2, PGK1 = Phosphoglycerate Kinase 1, PDIA3 = Protein disulfide-isomerase A3, TNFR = tumor necrosis factor receptor, FasL = Fas ligand, and TfR = Transferrin receptor.

EVs have garnered substantial attention in recent years as vital mediators of intercellular communication. These small, membrane-bound vesicles are secreted by various cell types into the extracellular environment, playing a central role in cell-to-cell signaling [13]. EVs can be categorized into several subtypes, with exosomes and microvesicles being the most extensively studied. Exosomes are small EVs, typically ranging from 30 to 150 nanometers in diameter. They originate from the endosomal system and are characterized by their unique lipid and protein composition [14]. Exosomes are formed within multivesicular bodies (MVBs), which contain intraluminal vesicles. Upon fusion of MVBs with the plasma membrane, exosomes are released into the extracellular space [15]. Microvesicles, also known as microparticles or ectosomes, are larger EVs, generally ranging from 100 to 1000 nanometers in size. Unlike exosomes, microvesicles are formed by the outward budding and shedding of the plasma membrane, resulting in the direct release of vesicles into the extracellular environment [16] (Figure 1).

The biogenesis of exosomes involves a tightly regulated process that begins with the formation of early endosomes. These early endosomes mature into MVBs, which contain intraluminal vesicles harboring specific cargo molecules. The cargo of exosomes encompasses a diverse array of bioactive molecules, including proteins, lipids, messenger RNA (mRNA), microRNA (miRNA), and other nucleic acids. Importantly, the composition of exosomal cargo is influenced by the originating cell type and its physiological state. Different mechanisms and factors are involved in the formation of exosomes, including Rab GTPases, subunits of the endosomal sorting complex required for transport (ESCRT), syntenin-1, tumor susceptibility gene 101 (TSG101), apoptosis-linked gene 2-interacting protein X (ALIX), ceramide, sphingomyelinases, and tetraspanins such as cluster of differentiation proteins CD9, CD63, and CD81 (Figure 1). These components also play a role in the regulation of cargo sorting into the vesicles. Microvesicles, on the other hand, encapsulate a distinct cargo that reflects the composition of the parent cell’s plasma membrane. This cargo may include adhesion molecules, surface receptors, and cytoplasmic proteins [17,18]. The formation of microvesicles partially depends on ESCRT proteins and the production of ceramide by sphingomyelinase. Moreover, the biogenesis of microvesicles requires the reorganization of protein and lipid components within the plasma membrane. This process includes the translocation of phosphatidylserine from the inner leaflet to the outer surface of the membrane, leading to physical membrane curvature and the reorganization of the actin cytoskeleton, ultimately resulting in membrane budding and vesicle release. A recent study identified proteins enriched in exosomes and microvesicles. Small extracellular vesicles (exosomes) were enriched with tetraspanins, ADAMs, ADAMTSs, and ESCRT proteins, as well as SNAREs and Rab proteins that are typically associated with endosomes. Conversely, large extracellular vesicles (microvesicles) were found to be enriched with ribosomal, mitochondrial, and nuclear proteins, along with proteins involved in cytokinesis. However, proteins such as Flotillin-1, most Rab proteins, and annexins did not show differential expression between the exosome and microvesicle subtypes [19].

EVs play pivotal roles in intercellular communication and are implicated in various physiological and pathological processes [20]. Their ability to transport bioactive molecules to target cells allows them to influence diverse cellular functions. Understanding the biogenesis and cargo composition of EVs is essential for elucidating their roles in intercellular communication and their significance in health and disease. In the immune system, EVs have emerged as crucial regulators of immune responses. Immune cells, including dendritic cells and macrophages, release EVs that are loaded with immunomodulatory molecules. These EVs can influence the behavior and function of neighboring immune cells, orchestrating immune responses [21]. In cancer biology, EVs derived from tumor cells contribute significantly to the tumor microenvironment. These tumor-derived EVs facilitate cancer progression by promoting cell proliferation, angiogenesis, and metastasis. They also play a role in immune evasion and drug resistance [22]. Within the nervous system, EVs have been recognized as key mediators of neuron–glia communication. These vesicles facilitate the transfer of bioactive molecules between neurons and glial cells, impacting synaptic plasticity, neuronal survival, myelination, microglial activation, and the overall function of the nervous system [23,24].

The isolation of EVs is a critical step in their study and application, with several techniques available to achieve this, such as differential ultracentrifugation, immune capture, ultrafiltration, and size exclusion chromatography [25]. Each method has its strengths and weaknesses, particularly in terms of the source of EVs, which can significantly affect the yield, purity, and biological relevance of the isolated vesicles. Table 1 provides a comparative analysis of common EV isolation techniques used in the studies mentioned in this review.

## 3. EVs as Mediators of Neuroinflammation

Neuroinflammation, a complex interplay of immune responses within the central nervous system (CNS), has emerged as a pivotal factor in the pathogenesis of various neurological disorders. This intricate process involves the activation of resident immune cells, such as microglia and astrocytes, along with the infiltration of peripheral immune cells. The dysregulation of these immune responses has been implicated in the progression of diseases ranging from neurodegenerative disorders to autoimmune conditions.

EVs play multifaceted roles in intercellular communication, with a particularly noteworthy impact on neuroinflammation, a pivotal process in various neurological disorders [24]. In the context of neuroinflammation, EVs serve as key mediators, facilitating the crosstalk between immune cells and neurons [106].

Microglia release EVs containing proinflammatory cytokines and chemokines. These EVs can propagate inflammatory signals to neighboring microglia and astrocytes, amplifying the neuroinflammatory response [34,51,107]. For instance, in conditions like multiple sclerosis (MS), which is a demyelinating disease, these EVs contain inflammatory molecules such as IL-1β, IFN-gamma, TNF, caspase 1, and the P2 × 7 receptor. They also carry metalloproteinases that, along with TNF and IL-1β, can disrupt the blood–brain barrier (BBB), promote degradation of the extracellular matrix, and facilitate the entry of immune cells into the brain. Similarly, activated astrocytes at the BBB release EVs containing IL-1β, worsening tissue damage and promoting cell death [62]. In MS patients, oligodendrocytes activate microglia, which further spreads the inflammatory response [108]. This is supported by findings showing increased levels of EVs in the blood and cerebrospinal fluid of acute MS patients. Injecting microglia-derived microvesicles into the brains of mice with an MS-like condition led to the recruitment of inflammatory cells to the site of injection [63]. These EVs also impact sphingosine metabolism in responsive cells. Knockout mice lacking a-SMase (sphingomyelinase), a key enzyme involved in EV production, show protection from MS-like conditions, indicating the importance of microvesicles in this disease [35,80].

Moreover, Toll-like receptors (TLRs), present in various cells of the nervous system including neurons, oligodendrocytes, astrocytes, and microglia, play a role in initiating inflammatory responses [109]. In the CNS, TLRs are activated by signals released from damaged or stressed cells, leading to tissue damage. EVs carrying altered miRNA content, particularly those involved in inflammation, are implicated in activating TLRs and initiating an inflammatory cascade in diseases like amyotrophic lateral sclerosis, Alzheimer’s disease (AD), Parkinson’s disease, and alcohol-induced brain damage [110,111].

In minimal hepatic encephalopathy (MHE), characterized by peripheral immune system alterations that impact the brain resulting in neuroinflammation, neurotransmission alterations, and cognitive and motor impairment [112,113,114,115], emerging evidence suggests EVs may contribute to the immune-mediated cerebral alterations [5,8,10]. MHE increased both the quantity and altered the protein cargo of EVs and the differentially expressed proteins were primarily associated with immune system processes. Moreover, injection of EVs from rats with MHE and hyperammonemia, but not from control rats, induced motor incoordination in recipient rats. This motor impairment was mediated by neuroinflammation, as evidenced by microglial and astrocytic activation, upregulation of IL-1β, TNFα and its receptor TNFR1, nuclear factor kappa B (NF-κB) in microglia, glutaminase I, and GAT3 in the cerebellum [5]. These findings suggest that plasma EVs from MHE carry molecules capable of triggering neuroinflammation in the cerebellum and mechanisms leading to motor incoordination.

Other emerging evidence suggests that EVs derived from mesenchymal stem cells (MSCs) possess anti-inflammatory properties and mitigate neuroinflammation in various pathological conditions. For instance, treatment with TGFβ-containing EVs derived from MSCs effectively activated TGFβ receptors, mitigating microglial activation and restoring the Smad7-IkB pathway to normal levels in MHE rats. Consequently, this inhibits NF-κB nuclear translocation in neurons, normalizes IL-1β expression, and restores membrane expression of AMPA and NMDA receptors, thereby improving cognitive function [10]. These findings highlight the therapeutic potential of MSC-derived EVs in ameliorating cognitive deficits associated with MHE-induced neuroinflammation. Importantly, the ability of MSC-derived EVs to restore cognitive function suggests their promising clinical utility in patients with MHE. However, further preclinical and clinical studies are warranted to validate the efficacy and safety of EV-based therapies in neurological diseases. Understanding the intricate roles of EVs in neuroinflammation is crucial for deciphering the pathophysiology of various neurological disorders and exploring potential therapeutic interventions targeting EV-mediated processes.

### 3.1. EVs in Glial Cell–Neuron Crosstalk

Within the CNS, communication between glial cells and neurons plays a pivotal role in various biological processes, encompassing brain development, neural circuit refinement, and the maintenance of homeostasis. Glial cells (astrocytes, oligodendrocytes, and microglia) not only orchestrate inflammatory reactions in response to infections or diseases but also continually provide neurotrophic support and contribute to synaptic remodeling and pruning. Apart from traditional direct cell-to-cell interactions and the paracrine effects of secreted molecules, glial cells, and neurons communicate via the release and uptake of EVs. This mode of communication enables coordinated regulation over long distances [12,106]. Notably, microglia, the innate immune cells of the CNS, heavily rely on mobile vesicles to disseminate cytokine-mediated inflammatory signals across distant brain regions [116].

#### 3.1.1. EVs from Microglia

Microglia release EVs that also play a crucial role in regulating synaptic transmission. These EVs influence neuronal function by increasing the production of two types of lipids, ceramide and sphingosine. This enhanced metabolism of sphingolipids has been shown to positively impact excitatory neurotransmission in both in vitro and in vivo experiments [36]. Microglia-derived EVs can interact with neurons and stimulate spontaneous and evoked excitatory transmission in vitro and after injection in vivo. Microglial-derived EVs appear to modulate synaptic activity and enhance neurotransmission [36,117].

Furthermore, recent studies have identified that inflammatory microglia release EVs containing an enrichment of microRNAs (miRNAs) capable of modulating the levels of synaptic proteins in recipient neurons. This phenomenon results in the loss of excitatory synapses, offering compelling evidence for a novel mechanism by which microglia-derived EVs may contribute to synaptic alterations in neurodegenerative processes [37]. In a model of mice with neuroinflammation it has been also shown that microglia are able to release cytosolic DNase in EVs, causing neuronal cell death [38]. The study further demonstrates that targeting microglial EVs to deliver recombinant DNase I can eliminate cytosolic double-stranded DNA, prevent neuroinflammation, reduce neuronal apoptosis, and delay neurodegenerative symptoms [38].

It has also been shown that polarized M1-microglia cells can induce apoptosis in neuronal PC12 cells through secreted EVs, and this regulatory effect may be mediated by different circular RNAs, a type of non-coding RNA with high stability in EVs [39]. Other authors have found that administering anesthesia and performing surgery promotes M1 polarization of microglia and the release of EVs with high expression of IL-1R1 [40]. These microglia-derived EVs then enhance IL-1R1 expression on the neuronal surface, facilitating binding to IL-1β which subsequently triggers activation of inflammatory signaling pathways within neurons leading to neuronal degeneration, synaptic loss, and ultimately postoperative cognitive dysfunction [40]. Contrarily, microglia in the M2 state release EVs rich in miR-672-5p, which inhibit the AIM2/ASC/caspase-1 signaling pathway. This inhibition leads to a reduction in neuronal pyroptosis and ultimately promotes the recovery of functional behavior in mice with traumatic spinal cord injury [41]. M2 microglial EVs attenuated BBB disruption after cerebral ischemia by delivering miR-23a-5p, which targeted TNF and regulated MMP3 and NFκB p65 expression [118].

Neuroinflammation also occurs early in Alzheimer’s disease, with microgliosis even preceding plaque formation, suggesting an unexpected pathological role for microglia in the first stages of the disease. In fact, it has been shown that amyloid-β released by microglia in association with EVs alters dendritic spine morphology in vitro, at the site of neuron interaction, and impairs synaptic plasticity both in vitro and in vivo in the entorhinal cortex–dentate gyrus circuitry [42]. 

Stress-triggered microglia secrete exosomes containing miR-146a-5p, which inhibits neurogenesis through the miR-146a-5p/KLF4 signaling pathway, contributing to depression [43]. Inflammatory stimuli can also upregulate miRNA-146a expression within neurons, mixed glial cells, and brain endothelial cells, which are either retained within these cells or released from them as EV cargo [51a]. The upregulation of miR-146a in EVs disrupts cellular bioenergetics, significantly reducing oxidative phosphorylation and glycolysis in glial cells [119].

**Figure 2:**
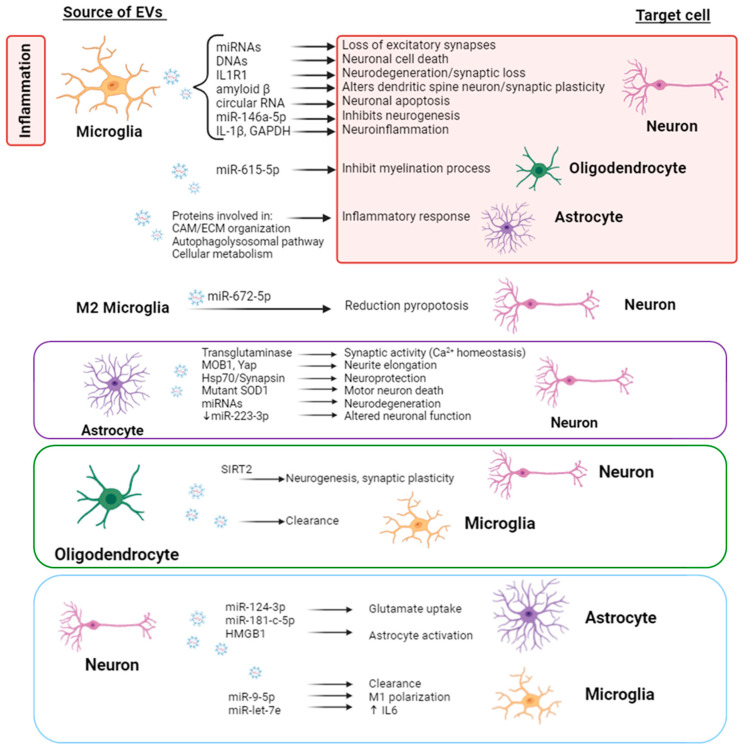
**EV-mediated intercellular crosstalk among glial-neuron cells.** EVs released by glial cells (astrocytes, oligodendrocytes, and microglia) or neurons have several target cells within the brain and not only orchestrate inflammatory reactions but also provide neurotrophic support and contribute to the maintenance of homeostasis. EVs from glial cells modulate synaptic activity, neuronal survival, neurogenesis, and myelination process, and, in an inflammatory environment, propagate the activation of inflammatory signaling pathways. Neuron-derived EVs also contribute to the homeostasis of astrocytes and microglia but, in neuroinflammatory conditions, they contribute to the activation of both. *CAM/ECM: cell adhesion/extracellular matrix*.

These findings support the idea that microglia can physiologically modulate synaptic activity, neuronal survival, and neurogenesis through the release of EVs, thus contributing to the fine-tuning of neural communication in the brain. In pathological conditions, microglia-derived EVs participate in the regulation and propagation of neuroinflammatory response (Figure 2).

EVs from activated microglia play a role in the process of demyelination and remyelination in the CNS by transferring miR-615–5p to oligodendrocyte precursor cells, inhibiting their maturation by targeting MYRF, a key transcription factor for myelination. This identifies miR-615–5p/MYRF as a potential therapeutic target for demyelinating diseases mediated by microglia-derived EVs [44].

Extracellular ATP has been recognized as a significant trigger for the release of vesicles from microglia, mediated by the activation of P2 × 7 receptors. Various studies have suggested that microglial-EVs induced by ATP are particularly rich in IL-1β and glyceraldehyde-3-phosphate dehydrogenase. This enrichment facilitates the spread of neuroinflammation within the brain [35,50]. It has also been demonstrated that ATP can induce alterations in the composition of microglia-derived EVs, resulting in an enrichment of proteins involved in cell adhesion/extracellular matrix organization, the autophagolysosomal pathway, and cellular metabolism. These changes subsequently influence the cellular response of astrocytes [45] (Figure 2).

#### 3.1.2. EVs from Astrocytes

In a manner akin to microglia, astrocytes release EVs in response to ATP-induced activation of P2 × 7 receptors, followed by the action of acid sphingomyelinase, as described by Bianco et al. [35]. These EVs derived from astrocytes have been attributed both beneficial and pathological roles. EVs with purported physiological functions harbor proteins implicated in neuroprotection, such as Hsp70 and synapsin I [46,52], as well as factors involved in angiogenesis regulation [47]. On the other hand, astrocytes expressing mutant SOD1 (copper–zinc superoxide dismutase) release elevated levels of EVs containing mutant SOD1, which can transfer to cultured neurons and induce motor neuron death, suggesting a role for EVs in amyotrophic lateral sclerosis pathogenesis [48]. In response to ATP or IL-10, astrocyte-derived EVs contain a set of proteins that enhance neurite outgrowth, dendritic branching, synaptic transmission, and neuronal survival. Conversely, astrocyte-derived EVs secreted in response to IL-1β contain proteins that regulate peripheral immune response and immune cell trafficking to the central nervous system [120]. Additionally, exposure of astrocytes to amyloid peptide triggers the release of pro-apoptotic EVs which are internalized by astrocytes and promote apoptosis, indicating that EV-mediated astrocyte demise may contribute to neurodegeneration in Alzheimer’s disease [53]. Neutral sphingomyelinase 2 (nSMase2) is an enzyme that generates the sphingolipid ceramide and plays a role in the regulation of neuroinflammation and cognition. Mice with nSMase2 deficiency show reduced levels of exosomes from astrocytes transporting miR-223–3p, which suppresses the expression of several genes important for neuronal function [54]. Moreover, the EV-mediated transfer of miRNAs from astrocytes to neurons has been proposed to participate in neurodegeneration in HIV-associated neurological disorders [49].

A recent study showed that activated astrocytes control intraneuronal Ca^2+^ levels by releasing transglutaminase-2 associated with EVs [55]. These astrocyte-derived EVs interact with neurons, increasing Ca^2+^ levels by inhibiting Na^+^/K^+^-ATPase. This inhibition leads to membrane depolarization and activation of an inward Ca^2+^ current through L-type Voltage Operated Calcium Channels and the Na^+^/Ca^2+^ exchanger, resulting in intracellular Ca^2+^ accumulation and Ca^2+^ dyshomeostasis. These changes could significantly impact synaptic activity during brain inflammation. 

Whether astrocyte-derived EVs interact with neurons at preferential sites and how EVs reach those sites on neurons remain elusive. D’Arrigo et al. (2021) demonstrated that astrocytes-derived EVs scan the neuron surface and use neuronal processes as highways to move extracellularly. The motion of EVs along neurites is facilitated by their binding to a surface receptor that moves along the neuronal membrane, facilitated by rearrangements of the actin cytoskeleton [121]. Extracellular motion may be a common feature among EVs from various cell types, including immune cells, suggesting a general role in propagating pathophysiological signals.

After spinal cord injury, a local inflammatory microenvironment is formed that not only exacerbates the secondary damage to neurons but also leads to the activation of resting astrocytes. Some studies have confirmed that activation of astrocytes is an adaptive change in the CNS in response to inflammatory damage and may play a positive role in facilitating neurite elongation. Sun et al. [56] showed that EVs from astrocytes can promote neurite elongation and motor function recovery in spinal cord injury model rats. This effect was enhanced when astrocytes were stimulated by LPS, promoting monopolar spindle binding protein 1 (MOB1) expression and reducing Yes-associated protein (YAP) levels [56] (Figure 2).

#### 3.1.3. EVs from Neurons

Men et al. [26] showed that secreted neuronal EVs contain a subset of miRNAs that is distinct from the miRNAs profile of neurons. These miRNAs, especially the neuron-specific miR-124-3p, are potentially internalized into astrocytes. MiR-124–3p further up-regulates the predominant glutamate transporter GLT1 by suppressing GLT1-inhibiting miRNAs. Another study demonstrated that epileptogenic neuronal EVs carrying miR-181c-5p decreased astrocyte glutamate uptake, thus increasing susceptibility to epilepsy [27]. 

It has also been shown that high mobility group box 1 (HMGB1), a factor capable of initiating an inflammatory signaling cascade, is primarily released within EVs from stressed neurons, which are taken up by surrounding astrocyte processes [31]. This facilitates selective communication between neurons and astrocytes while bypassing microglia, as demonstrated by the activation of the proinflammatory transcription factor NF-ĸB p65 in astrocytes but not in microglia [31]. This study indicates that proinflammatory mediators released within EVs from neurons can trigger cell-specific inflammatory signaling in astrocytes without activating transmembrane receptors on other cells and causing widespread inflammation.

This highlights a novel mechanism of intercellular communication in the central nervous system, shedding light on how neuronal signals regulate astroglial functions.

Other evidence also suggests that EVs originating from neurons and oligodendrocytes can regulate microglial activity functionally. EVs released by neurons, a process heightened by potassium-induced depolarization, have been demonstrated to enhance microglial clearance of degenerating neurites by inducing an increase in the expression of the complement molecule C3 in microglia [28,33]. Similarly, EVs originating from oligodendrocytes, the myelin-forming cells of the CNS, are internalized by microglia through macropinocytosis and subsequently transported to lysosomes for functional degradation [57,116]. Further investigations have revealed that this process neither impacts cellular motility nor alters the cytokine expression profile of microglia, suggesting that microglia possess a specialized mechanism for clearing oligodendroglial membranes in an immunologically ‘silent’ manner, as proposed by Fitzner et al. [58]. It has also been shown that EVs from neurons can alter microglial polarization by transferring miR-9-5p, leading to M1 polarization of microglia and subsequent neuronal injury [29]. This crosstalk between neurons and microglia, mediated by EVs, promotes the release of inflammatory factors by suppressing SOCS2 expression and activating the JAK/STAT3 pathways [29]. Other authors found that IL-6 gene expression was increased in human microglia after treatment with neuron-derived exosomes of AD patients with a high amount of miRNA let-7e, indicating once again the involvement of EVs in this neuron–microglia crosstalk [32] (Figure 2).

#### 3.1.4. EVs from Oligodendrocytes

It has also been shown that EVs secreted by oligodendrocytes transport cargo to neurons and may contribute to axonal integrity [59,122]. Mice lacking genes encoding oligodendroglial proteolipid protein and 20,30-cyclic nucleotide 30-phosphodiesterase develop secondary progressive axonal degeneration characterized by the formation of axonal swellings. Mutant oligodendrocytes release fewer exosomes, which are unable to support neurons deprived of nutrients and facilitate axonal transport [122]. The release of EVs from oligodendrocytes is regulated by neurotransmitter signaling. When neurons are active, they release glutamate, which activates ionotropic glutamate receptors on oligodendrocytes, primarily of the NMDA subtype, leading to an influx of calcium ions and subsequent EV secretion. Neurons selectively internalize EVs derived from oligodendrocytes, while astrocytes and other oligodendrocytes show minimal uptake. Once internalized by neurons, the cargo carried by oligodendroglial EVs can exert functional effects. Additionally, oligodendroglial EVs have been found to enhance the metabolic activity of neurons cultured under cellular stress conditions. This suggests a model in which active neurons communicate with oligodendrocytes, requesting the delivery of supportive biomolecules via EVs. Oligodendrocytes then utilize these vesicles to locally transfer metabolites, protective proteins, glycolytic enzymes, mRNA, and miRNA to axons, thereby potentially preserving axonal integrity. The transfer of EVs from oligodendrocytes to neurons in response to neurotransmitter signaling suggests that these vesicles may mediate glial support of neurons. Other authors have shown that oligodendrocyte-derived EVs enriched with SIRT2 reduce behaviors resembling depression and enhance neurogenesis and synaptic plasticity in the hippocampus following chronic unpredictable mild stress in mice [60]. The positive effects of oligodendrocyte-derived EVs appear to be attributed to the delivery of SIRT2 and the activation of the AKT/GSK-3β signaling pathway, which regulates neuroplasticity [60] (Figure 2).

In the peripheral nervous system, recent research has shown that neuronal activity enhances the release of EVs from regenerative Schwann cells (rSCs) and their transfer to neurons [123]. This process is mediated by the activation of P2Y receptors in Schwann cells following activity-dependent ATP release from sensory neurons. Crucially, activating P2Y receptors in rSCs also increases the amount of miRNA-21 in the EVs derived from these cells [123]. Overall, the findings demonstrate that communication between neurons and glial cells through ATP-P2Y signaling regulates the content of Schwann cell-derived EVs and their transfer to axons. This modulates axonal elongation in a non-cell autonomous manner, indicating that axonal growth is influenced by external signals and vesicular content from Schwann cells in response to neuronal activity.

All these studies have attributed both pathological and physiological functions to glial EVs, including the spread of pathogenic factors, promotion of inflammation, modulation of neurotransmission, and support of neuronal function (Figure 2).

### 3.2. EVs at CNS Barrier

The peripheral immune system exerts significant influences on brain functions, and communication from the periphery to the brain has been implicated in the pathophysiology of various CNS disorders. For instance, systemic inflammation has been proposed to be transmitted to the brain, inducing neuroinflammation that alters neurotransmission, leading to neuronal dysfunction associated with inflammation [2,124]. Additionally, there is evidence supporting the involvement of EV-mediated communication from the periphery to the brain during inflammatory conditions [65]. However, the mechanisms through which systemic inflammation transmits inflammatory signals to the brain via EVs remain poorly understood. 

The CNS is shielded by barriers that create a division between the bloodstream and the brain tissue along with the cerebrospinal fluid (CSF), the blood–brain barrier (BBB), and the blood–CSF barrier (BCB). The BBB is the brain’s primary defense barrier, composed of specialized endothelial cells called brain microvascular endothelial cells (BMECs). These cells tightly adhere to each other through tight junctions and adherens junctions, restricting the passage of small molecules between them. Together with pericytes and astrocytic endfeet, they form the neurovascular unit, contributing to maintaining the BBB’s barrier function [125]. The BCB is comprised of epithelial cells located in the choroid plexus, extending into the brain’s ventricles and tasked with producing CSF. These choroid plexus epithelial cells are tightly connected via tight junctions and are oriented towards the CSF at their apical side, while blood vessels adjacent to them possess a fenestrated endothelium, facilitating the exchange of molecules at their basal side [126].

EVs have three main modes of interaction with CNS barriers [12]: (1) Barrier cells can release EVs, which can act locally within the barrier environment, penetrate the brain tissue, or enter the bloodstream, carrying their cargo to distant sites. (2) EVs originating from the brain or circulating in the bloodstream can communicate with CNS barriers, influencing their function and characteristics. (3) EVs can cross the barrier either nonspecifically during disease-induced barrier breakdown or through a selective transportation process facilitated by surface molecules, enabling entry via specific mechanisms.

The release of CNS-derived EVs into the bloodstream is mainly observed in pathological situations characterized by barrier disruption, such as traumatic injury, brain tumors, or neurodegenerative diseases [66,67,68,69]. Likewise, the infiltration of peripheral EVs into CNS tissues is facilitated by inflammatory responses.

Entry of EVs into the CNS seems to be selective, involving various receptors found on both the endothelial cells of the brain and the membranes of EVs themselves. Several studies, both in vitro and in vivo, indicate that EVs are transported via adsorptive transcytosis through different endocytic transcellular pathways dependent on dynamin, clathrin, and caveolin [64,107]. CD46, transferrin receptor, C-type lectin receptors, and heparan sulfate proteoglycan have been suggested as being present on the surface BMECs and involved in facilitating the entry of EVs [61,72,78,84].

In general, the ability of EVs to cross the BBB has been demonstrated in various experimental models, both in vivo and in vitro, with inflammation often facilitating this process. While EVs from different sources seem to have the capacity to enter the brain, the specific mechanism and speed of entry depend on the unique characteristics of the EVs and the surrounding environment, particularly the presence of inflammation. Whether EVs can traverse the blood–CSF barrier remains uncertain, but there is no inherent reason to believe that it cannot be breached using mechanisms similar to those observed for the BBB.

It has been shown that systemic inflammation triggers an increase in the release of choroid plexus epithelial cell-derived extracellular vesicles (CPEC-EVs), which in turn induce a pro-inflammatory response in astrocytes and microglia through the miRNA cargo carried by these EVs. Upon reaching the brain parenchyma, CPEC-EVs within the CSF activate an inflammatory response [79]. In Alzheimer’s disease model mice, levels of CPEC-EVs are elevated in the CSF, and inhibiting the release of EVs prevents cognitive decline induced by amyloid-beta [77], highlighting the significance of this CPEC–EV signaling pathway in neurodegenerative disorders.

This suggests the possibility that systemic inflammation induces the release of inflammatory EVs by peripheral immune cells, which may directly traverse the BBB to target brain cells. Alternatively, intermediary cells such as epithelial cells may be required to respond to the systemic environment, including circulating inflammatory EVs, by releasing their own EVs. Once within the parenchyma, EVs derived from epithelial cells can be internalized by various brain cell types, including microglia, astrocytes, and neurons [65,74,79]. Further investigations are necessary to elucidate the mechanisms by which EVs transmit systemic signals to the brain under both physiological and pathological conditions.

Despite challenges associated with methodologies for studying EVs and the complex morphology of CNS barriers, significant strides have been made through a combination of in vitro cell studies, in vivo modeling, and genetic approaches. However, technical limitations remain, such as difficulties in accurately labeling EVs with dyes, ensuring the purity and classification of EV particles, and developing methods to selectively inhibit EV release without affecting all EV subtypes or causing unintended effects [127]. Awareness of these limitations is crucial for designing rigorous experiments and interpreting results accurately, as well as for driving future innovations to overcome these challenges.

## 4. EVs in Interactions between Different Organs and Neuroinflammation

### 4.1. EVs in Brain–Heart Axis

The role of EVs in heart-brain crosstalk has only recently gained attention in the last years [128]. Cardiac dysfunction has been linked to an increased risk of stroke [129]. Nearly half of patients undergoing cardiac surgery exhibit heightened permeability of the BBB, a phenomenon also observed in stroke patients [130]. EVs have the ability to cross the BBB bidirectionally under normal physiological conditions, a process that is further enhanced following barrier damage. It has been shown that plasma astrocyte-derived EVs remained significantly elevated 5–30 days post-ischemic stroke [131]. The increased levels of circulating brain-derived EVs associated with stroke may contribute to cardiac dysfunction induced by brain damage [132]. These EVs disrupt endothelial function by inhibiting the synthesis of nitric oxide (NO) through the inhibition of endothelial NO synthase and by increasing the levels of caveolin-1 [132]. The EV membrane contains procoagulant factors such as phosphatidylserine and tissue factor [71,85]. EVs have the ability to attach to coagulation factors and stimulate their activation. EVs contain P-selectin glycoprotein ligand-1, which binds to P-selectin molecules exposed by endothelial cells or platelets when vascular damage occurs. This interaction activates tissue factor, initiating the process of thrombosis [133].

These mechanisms could help elucidate the findings of the clinical trial PROSCIS-B (Prospective Cohort With Incident Stroke Berlin). This study observed a link between elevated levels of microvesicles derived from leukocytes and endothelial cells following a stroke and poorer cardiovascular outcomes within a three-year period [70].

The EV content, such as miRNAs, has been linked to both heart and brain pathophysiology [134]. Reduced circulating levels of miR-126 have been reported in stroke patients, and the authors of this study suggested that they may be carried by EVs and impact other organs, such as the heart [135]. MiR-126, also found in neuronal EVs, is important for heart function [30]; knockout mice lacking endothelial cell-derived miR-126 had worse heart issues after stroke [136]. MiR-126 levels are low in heart failure and atrial fibrillation [137]. Another key miRNA, miR-210, transported by mesenchymal stem cell-derived EVs, promotes blood vessel formation in the brain and heart [138,139]. MiR-210 increases the levels of hepatocyte growth factor, aiding in blood vessel formation, brain cell growth, and nerve connections [140]. The miR-17~92 family of miRNAs clusters, carried by mesenchymal stem cell-derived EVs [141], controls the proliferation regulator of cardiomyocytes and of neural progenitor cells after stroke [142,143]. It also protects the heart and brain during ischemia by targeting the PI3K/AKT and the MAPK/ERK-specific cellular pathways [134].

Serum extracellular vesicle-derived miR-124–3p, mainly found in the brain and known for activating protective pathways such as PI3K/AKT and MAPK/ERK, showed a significant decrease within 24 h after stroke and was inversely related to the size of the stroke-induced tissue damage [76,144]. Similarly, the level of miR-124 carried by circulating EVs decreased during acute ischemic stroke. In the case of the heart, miR-124 levels rise notably during myocardial infarction, and blocking this rise reduces cardiomyocyte apoptosis [145].

**Figure 3:**
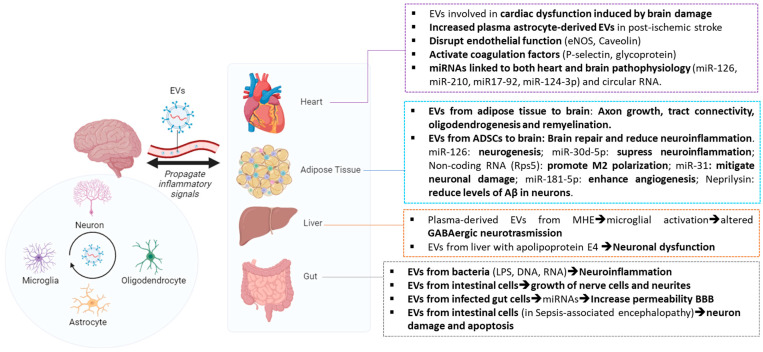
**Scheme of EVs in inter-organ crosstalk.** EVs disseminate inflammatory signals between organs. In brain–heart crosstalk, increased plasma astrocyte-derived EVs have been shown in post-ischemic stroke. The content of the EVs participates in the disruption of endothelial function and activation of coagulation factors. Moreover, several miRNAs have been found to be linked to both heart and brain pathophysiology. EVs from adipose tissue are involved in processes such as axonal growth, tract connectivity, oligodendrogenesis, and remyelination following subcortical ischemic stroke. Other emerging evidence suggests that EVs derived from mesenchymal stem cells, such as ADSCs (adipose-derived stem cells), possess anti-inflammatory properties and mitigate neuroinflammation in various pathological conditions. Plasma EVs from animal models with minimal hepatic encephalopathy (MHE) are able to induce altered neurotransmission. Age-related thyroid deficiency can enhance the transport of Apolipoprotein E4-containing EVs from the liver to the brain, contributing to Alzheimer’s disease-related dementia and neuronal dysfunction. In the gut–brain axis, EVs found in the intestinal microenvironment originate from both microorganisms, such as bacteria, and intestinal cells, and are involved in transmitting signals (LPS, DNA, RNA, miRNAs, etc.) to the brain through the vagus nerve or the bloodstream. These gut-derived EVs can induce neuroinflammation, modulate neuronal function, and increase the permeability of the blood–brain barrier (BBB).

Changes in circular ribonucleic acid expression have also been shown within EVs from the brain of mice with traumatic brain injury and cardiac muscle contraction and calcium signaling were functions affected by circular ribonucleic acid [146]. This indicates that circular RNA carried by EVs could play a role in facilitating communication between the brain and heart (Figure 3).

### 4.2. EVs in Brain–Adipose Tissue Axis

There is evidence indicating that EVs play a role in facilitating communication between the brain and adipose tissue. Adipose tissue generates numerous biologically active substances that interact with peripheral organs and the CNS [147]. Adipokines, released by adipose tissue, regulate neuroinflammation and oxidative stress—key physiological and pathophysiological processes in the CNS—and are associated with various CNS disorders. Adipose tissue is also a significant origin of circulating non-coding RNAs, many of which are carried by EVs. In fact, some authors have proposed that adipose tissue is the primary contributor to circulating EVs [148].

It has been shown that EVs derived from adipose tissue are involved in processes such as axonal sprouting, tract connectivity, oligodendrogenesis, and remyelination following subcortical ischemic stroke. Adipose tissue contains adipose-derived stem cells (ADSCs), an important source of EVs [86]. A proteomic analysis of EVs from ADSCs identified over 2000 proteins associated with brain repair, suggesting that EVs could potentially enhance functional recovery [86].

EVs derived from ADSCs containing high levels of miR-126 can mitigate the effects of ischemic stroke [91]. These EVs inhibit the activation of microglia and inflammatory responses triggered by ischemic stroke while promoting neurogenesis and functional recovery. Additionally, ADSC-derived EVs rich in miR-30d-5p protect against acute ischemic stroke by inhibiting autophagy-mediated polarization of microglia/macrophages, thereby suppressing inflammation and reducing the extent of brain damage caused by infarction [87].

EVs derived from ADSCs that were pre-treated with hypoxia have been shown to reduce brain damage caused by acute ischemic stroke and promote polarization of M2 microglia/macrophages. They achieve this by delivering a non-coding RNA called circ-Rps5 [94]. Some authors suggested other molecules involved in EV-mediated communication between adipose tissue and neurons. Specifically, EVs carrying miR-31 target neurons and downregulate TRAF6, leading to the upregulation of interferon regulatory factor 5 and mitigating neuronal damage caused by ischemic stroke [88]. ADSC-derived EVs protect neurons by inhibiting the NF-κB and mitogen-activated protein kinase (MAPK) pathways and preventing microglia activation [89]. ADSC-derived EVs enhance the angiogenesis of brain microvascular endothelial cells after oxygen–glucose deprivation (OGD) via miR-181b-5p, suggesting a novel role for these EVs in stroke recovery [93].

Studies have investigated the link between cognitive decline and EVs derived from adipose tissue. It has been shown that adipose tissue-derived EVs from high-fat diet-fed mice contribute to cognitive impairment by causing synaptic loss and neuroinflammation, thus exacerbating cognitive decline. Furthermore, blocking miR-9–3p in adipose tissue-derived EVs may prevent cognitive impairment associated with insulin resistance in obesity [82]. Alzheimer’s disease (AD) is characterized by the accumulation of amyloid-beta (Aβ) in the brain. ADSC-derived EVs containing active neprilysin have been shown to reduce levels of Aβ in a neuronal cell line with overexpressed Aβ, suggesting a potential therapeutic approach for AD [90]. In vitro studies have demonstrated that ADSC-derived EVs can alter the cellular phenotype of amyotrophic lateral sclerosis (ALS), including reducing aggregation of superoxide dismutase 1 and improving mitochondrial function, indicating their potential therapeutic use in ALS [92]. It has also been reported that ADSC-derived EVs reduce neuroinflammation in the hippocampus and restore cognitive function in chronic hyperammonemia, a main contributor to hepatic encephalopathy [10]. Therefore, although ADSC-derived EVs hold promise for restoring brain function, further research is needed to explore their therapeutic potential fully (Figure 3).

### 4.3. EVs in Brain–Liver Axis

Hepatic encephalopathy is a neuropsychiatric syndrome that highlights the intricate connection between the liver and the brain [2]. Cirrhotic patients often exhibit persistent hyperammonemia and neuroinflammation, leading to moderate neurological symptoms such as motor incoordination and mild cognitive impairment, a condition known as minimal hepatic encephalopathy [149]. Chronic hyperammonemic rats develop neurological alterations including neuroinflammation in the cerebellum and hippocampus, disrupted GABAergic and glutamatergic neurotransmission, and deficits in motor coordination and cognitive function [112,150,151,152,153,154].

It has been shown that EVs play a role in transmitting changes from the periphery to the brain in minimal hepatic encephalopathy. In hyperammonemic rats, plasma EVs underwent alterations and showed increased levels of TNF-α. When these EVs from hyperammonemic rats were injected into normal rats, they reached the cerebellum and induced changes in Purkinje neurons and microglia, leading to neuroinflammation and motor incoordination [5]. Moreover, the elevated TNF-α levels in EVs from hyperammonemic rats activated various pathways in the cerebellum, including the TNFα-TNFR1-NF-κB-glutaminase-GAT3 pathway, which enhanced GABAergic neurotransmission. This resulted in microglia activation, increased GABAergic neurotransmission, and subsequent motor coordination problems [5].

Age-related thyroid deficiency can enhance the transport of Apolipoprotein E4-containing EVs from the liver to the brain, contributing to Alzheimer’s disease-related dementia and neuronal dysfunction [73]. These changes are accompanied by the activation of the NLRP3 inflammasome and neuronal pyroptosis induced by ApoE4 [73]. Additionally, EVs isolated from the serum of rats with hepatic ischemia-reperfusion injury may cause neuronal damage in the hippocampus and cortex, involving the NLRP3 inflammasome and caspase-1-dependent pyroptosis [75]. The role of EVs in brain–liver communication under normal and pathological conditions holds promise for future investigation (Figure 3).

### 4.4. EVs in Brain–Gut Axis

Disruptions in the gut environment have been linked to several neuropsychiatric and neurological disorders [155]. The gut–brain axis comprises various signaling pathways that transmit signals to the CNS through the vagus nerve or the bloodstream. However, the role of EVs in facilitating communication between the brain and gut has only recently begun to receive attention [156].

EVs found in the intestinal microenvironment originate from both microorganisms, such as bacteria and fungi, and intestinal cells. Bacteria can release EVs, which play crucial roles in mediating interactions between microbes and their host and are involved in immune functions and disease development [157].

It has been shown that bacterial EVs can cross the BBB and deliver their cargo [158].

Lipopolysaccharide [LPS], found in bacterial EVs, has been associated with neuroinflammation. These EVs can increase BBB permeability, activate astrocytes and microglia, trigger inflammatory responses, and induce tau hyperphosphorylation via the glycogen synthase kinase-3 beta (GSK-3β) pathway, ultimately leading to cognitive impairment [95]. These neural effects resemble those observed in Alzheimer’s disease pathology. Authors suggest that bacteria-derived EVs may exploit the vagus nerve and bloodstream to facilitate communication between the gut and brain [96]. This suggests that bacterial EV transport to the brain could be linked to infections occurring anywhere in the body, potentially resulting in neuroinflammation. Many neurodegenerative diseases are linked to neuroinflammation and changes in microbiome composition. In such cases, bacterial EVs can transport lipopolysaccharides, DNA, and RNA to the brain, triggering neuroinflammation and influencing gene expression [159]. For instance, in mice, bacterial EVs can cross the BBB and activate inflammatory signaling pathways like TLR8 and NF-κB, or activate IL-6 and NF-κB in brain monocytes and microglia, contributing to neuroinflammatory diseases such as Alzheimer’s disease [100,101]. 

Interestingly, it has also been shown that enteric bacterial EVs carry neurotransmitters such as GABA and glutamate that have an impact on the brain [97], highlighting the need for more research to understand their effects on neurodegenerative diseases. Furthermore, gut microorganism-derived EVs can positively influence the brain by, for example, increasing BDNF levels in hippocampal cells and alleviating stress-induced depression-like behaviors or inducing antidepressant effects by restoring hippocampal neurotrophic factors [98,99].

However, it has also been reported that EVs released by intestinal cells play a role in brain function. Studies have shown that intestinal epithelial cell-derived EVs treated with GABA promote the growth of nerve cells and neurites [102]. Factors like intestinal microbial infections and toxins can also impact the composition of gut-derived EVs, potentially affecting distant organs [105]. For instance, gut cells infected with *Escherichia coli* release EVs containing miRNAs associated with increased permeability of the blood–brain barrier [103]. The immune system in the gut can be activated by EVs from intestinal epithelial cells containing peptides complexed with MHCII, which may contribute to neuroinflammation and brain damage [83]. In rats with sepsis-associated encephalopathy, disturbances in intestinal flora led to increased release of intestinal epithelium-derived EVs, causing hippocampal neuronal damage and apoptosis, an effect that could be mitigated by inhibiting EV release [104] (Figure 3).

## 5. Conclusions

Recent research has shed light on the involvement of EVs in neuroinflammation, highlighting their role in propagating inflammatory signals and influencing disease progression. EVs, released by diverse cell types within the nervous system, carry specific cargo, including nucleic acids, proteins, and lipids, capable of regulating immune responses and glial and neuronal functions. There is a need for greater uniformity in the methods of EV isolation and characterization. Enhancing in vivo isolation methods for EVs will undoubtedly facilitate the discovery of novel biological functions. While many EV studies have been performed using in vitro cell cultures, further investigations involving animal and clinical research will be a key to unlocking the full potential of EV biology.

EVs derived from glial cells not only propagate inflammation in response to infections or diseases but also provide neurotrophic support, contributing to synaptic activity, neuronal survival, neurogenesis, demyelination, and remyelination processes in the CNS. Neuron-derived EVs also contribute to the homeostasis of astrocytes and microglia but, under neuroinflammatory conditions, they activate both. This mode of communication through EVs enables coordinated regulation over long distances within the brain and also between organs. While EVs from different sources seem to have the capacity to enter the brain, the specific mechanism and speed of entry depend on the unique characteristics of the EVs and the surrounding environment, particularly the presence of inflammation. Further comprehensive investigation is required to elucidate these mechanisms fully.

Bidirectional communication networks between the brain and other organs, such as the heart, adipose tissue, liver, and gut, are emerging, with EVs playing a significant role in disseminating inflammatory signals between organs. Additionally, emerging evidence suggests that EVs derived from mesenchymal stem cells (MSCs), such as adipose-derived stem cells, possess anti-inflammatory properties and mitigate neuroinflammation in various pathological conditions. The potential clinical utility of MSC-derived EVs in restoring cognitive function is promising, yet further preclinical and clinical studies are warranted to validate the efficacy and safety of EV-based therapies in neurological diseases.

Understanding the intricate roles of EVs in neuroinflammation is crucial for deciphering the pathophysiology of neurological disorders and exploring potential therapeutic interventions targeting EV-mediated processes. Recognizing EVs as a signaling system that transcends boundaries offers valuable insights and holds promise for more effective biological therapies. However, further research is required to fully comprehend the mechanisms by which EVs enable communication between the brain and other organs. As our understanding of this cross-border signaling mechanism grows, there is immense potential for addressing unresolved questions in this field. 

## Figures and Tables

**Table 1:** Comparison of EV isolation techniques in terms of the origin source.

Source of EVs	Methodology to Isolate EVs	References
Neuron	Ultracentrifugation	Men Y et al. (2019) [26] Ma L et al. (2024) [27] Bahrini I, et al. (2015) [28] Xian X et al. (2022) [29] Wang H et al. (2021) [30]
Neuron	Exosome isolation Kit Immunocapture anti-CD171	Kaya Z et al. (2023) [31] Durur DY et al. (2022) [32]
Cortical neurons	Ultracentrifugation Size-exclusion chromatography	Solana-Balaguer J et al. (2023) [23]
Cortical neurons	Ultracentrifugation/Sucrose gradient	Fauré J et al. (2006) [33]
Astrocytes, Microglia	Ultracentrifugation	Yang Y et al. (2018) [34] Bianco F, et al. (2009) [35] Antonucci F, et al. (2012) [36] Prada I, et al. (2018) [37] Arvanitaki ES et al. (2024) [38] Gao S et al. (2022) [39] Qi Z et al. (2023) [40] Zhou Z et al. (2022) [41] Gabrielli M et al. (2023) [42] Fan C et al. (2021) [43] Ji XY et al. (2024) [44] Drago F, et al. (2017) [45] Taylor, A. R., et al. (2007) [46] Hajrasouliha, A. R., et al. (2013) [47] Basso, M., et al. (2013) [48] Hu, G., et al. (2012) [49]
Microglia	Exosome Isolation Reagent	Takenouchi T, et al. (2015) [50]
Murine microglial cell	Centrifugation	La Torre ME et al. (2022) [51]
Astrocytes	Ultracentrifugation/Sucrose gradient	Wang, S., et al. (2011) [52]
Astrocytes	Ultracentrifugation. Precipitation method (ExoQuick solution)	Wang, G., et al. (2012) [53]
Astrocytes	Centrifugation Membrane-based affinity columns (exoEasy kit)	Zhu Z et al. (2022) [54]
Astrocytes	Ultracentrifugation	Tonoli E et al. (2022) [55] Sun H et al. (2022) [56]
Oligodendrocytes	Ultracentrifugation/Sucrose gradient	Krämer-Albers EM, et al. (2007) [57] Fitzner D, et al. (2011) [58] Frühbeis, C., et al. (2013) [59]
Oligodendrocytes	Immunoprecipitation (ExoQuick/anti-CNPase)	Zhang H et al. (2024) [60]
Macrophage	Ultracentrifugation	Yuan D et al., 2017 [61]
Human fluids and culture media	Ultracentrifugation. Sucrose gradient combined with centrifugation. Polymeric precipitation.	Sáenz-Cuesta M et al. (2014) [62]
Platelets, leukocytes, or monocytes	Flow cytometry	Saenz-Cuesta M, et al. (2014) [63]
Erytrocytes	Ultracentrifugation/Sepharose CL-2B	Matsumoto J, et al. (2017) [64]
Hematopoietic cell culture Plasma/Serum	Ultracentrifugation/Sucrose gradient	Ridder K, et al. (2018) [65]
Plasma	Size exclusion chromatography	Schindler CR et al. (2024) [66]
Plasma	Ultracentrifugation	Ricklefs FL et al. (2024) [67]
Plasma	Precipitation method (ExoQuick solution)	lommer J, et al. (2022) [68] Mustapic M, et al. (2017) [69]
Plasma	Flow cytometry	Huo S et al. (2021) [70]
Platelet-free plasma	Centrifugation	Hisada Y et al. (2019) [71]
Serum	Ultracentrifugation	Qu M, et al. (2018) [72] Zhang M et al. (2022) [73]
Serum	Precipitation method (ExoQuick solution) Ultracentrifugation	Li JJ, et al. (2018) [74] Zhang L et al. (2019) [75]
Serum	Precipitation method kit	Qi Z et al. (2021) [76]
Breast cancer cell line	Differential ultracentrifugation Density gradient centrifugation	Lischnig A et al. (2022) [19]
Breast cancer cell line	Ultracentrifugation	Morad G et al. (2019) [77]
Melanoma cell line	Precipitation method (ExoQuick solution)	Kuroda H et al. (2019) [78]
Human mast cell line	Precipitation and differential ultracentrifugation Size exclusion chromatography Immunocapture and density gradients	Pfeiffer, A et al. (2022) [16]
CSF ^1^ and cell culture	Precipitation method	Balusu S, et al. (2016) [79]
Rodent CSF ^1^	Ultracentrifugation	Verderio C, et al. (2012) [80]
CPE ^2^ cultures	Size exclusion chromatography	Vandendriessche C, et al. (2021) [81]
Adipose tissue	Ultracentrifugation	Wang J et al. (2022) [82]
Liver	Ultracentrifugation	Zhang M et al. (2022) [73]
Mesenteric lymph	Ultracentrifugation	Kojima M et al. (2018) [83]
Neural stem cells	Ultracentrifugation	Joshi BS et al. (2021) [84]
Human Bone Marrow MSC ^3^	Ultracentrifugation	Wei X et al. (2016) [85]
MSC ^3^	Exosome extraction kit (miRCURYTM Exosome Isolation Kit, EXIQON)	Otero-Ortega L et al. (2017) [86]
Adipose derived SC ^4^	Ultracentrifugation	Jiang Mcet al (2018) [87] Lv H et al. (2021) [88] Feng N et al. (2019) [89] Katsuda T et al. (2013) [90]
ADSC ^5^	Precipitation method (ExoQuick solution)	Geng W et al. (2019) [91] Lee M et al. (2016) [92]
ADSC ^5^	Exosome Isolation kit	Yang Y et al. (2018) [93]
ADSC ^5^	Centrifugation/Ultra-15 Centrifugal Filter	Yang H et al. (2022) [94]
Faeces	Ultracentrifugation	Wei S et al. (2020) [95]
Bacteria	Ultracentrifugation	Lee KE et al. (2020) [96] Zakharzhevskaya NB et al. (2017) [97] Choi J et al. (2019) [98] Choi J et al. (2022) [99]
Bacteria	ExoBacteria OMV Isolation Kit	Ha JY et al. (2020) [100] Han EC et al. (2019) [101]
Intestinal cells	Exosome Isolation Kit	Inotsuka R et al. (2020) [102] Larabi A et al. (2020 [103] Xi S et al. (2021) [104]
Intestinal cells	Ultracentrifugation, density gradient separation, and polymer-based precipitation methods	Ayyar KK et al. (2021) [105]

^1.^ CSF: Cerebrospinal fluid; ^2^ CPE: Choroid Plexus explant; ^3^ MSC: Mesenchymal Stem Cells; ^4^ SC: stem cell; ^5^ ADSC: Adipose derived stem cells.

## References

[1] Heneka M.T., Kummer M.P., Latz E. (2014). Innate immune activation in neurodegenerative disease. Nat. Rev. Immunol..

[2] Cabrera-Pastor A., Llansola M., Montoliu C., Malaguarnera M., Balzano T., Taoro-Gonzalez L., García-García R., Mangas-Losada A., Izquierdo-Altarejos P., Arenas Y.M. (2019). Peripheral inflammation induces neuroinflammation that alters neurotransmission and cognitive and motor function in hepatic encephalopathy: Underlying mechanisms and therapeutic implications. Acta Physiol..

[3] Prinz M., Priller J. (2014). Microglia and brain macrophages in the molecular age: From origin to neuropsychiatric disease. Nat. Rev. Neurosci..

[4] Gao H.M., Hong J.S. (2008). Why neurodegenerative diseases are progressive: Uncontrolled inflammation drives disease progression. Trends Immunol..

[5] Izquierdo-Altarejos P., Cabrera-Pastor A., Gonzalez-King H., Montoliu C., Felipo V. (2020). Extracellular Vesicles from Hyperammonemic Rats Induce Neuroinflammation and Motor Incoordination in Control Rats. Cells.

[6] Théry C., Witwer K.W., Aikawa E., Alcaraz M.J., Anderson J.D., Andriantsitohaina R., Antoniou A., Arab T., Archer F., Atkin-Smith G.K. (2018). Minimal information for studies of extracellular vesicles 2018 (MISEV2018): A position statement of the International Society for Extracellular Vesicles and update of the MISEV2014 guidelines. J. Extracell. Vesicles.

[7] Vella L.J., Hill A.F., Cheng L. (2016). Focus on Extracellular Vesicles: Exosomes and Their Role in Protein Trafficking and Biomarker Potential in Alzheimer’s and Parkinson’s Disease. Int. J. Mol. Sci..

[8] Gallego J.J., Fiorillo A., Casanova-Ferrer F., Urios A., Ballester M.P., Durbán L., Megías J., Rubio T., Cabrera-Pastor A., Escudero-García D. (2022). Plasma Extracellular Vesicles Play a Role in Immune System Modulation in Minimal Hepatic Encephalopathy. Int. J. Mol. Sci..

[9] Xiong Y., Mahmood A., Chopp M. (2017). Emerging potential of exosomes for treatment of traumatic brain injury. Neural Regen. Res..

[10] Izquierdo-Altarejos P., Cabrera-Pastor A., Martínez-García M., Sánchez-Huertas C., Hernández A., Moreno-Manzano V., Felipo V. (2023). Extracellular vesicles from mesenchymal stem cells reduce neuroinflammation in hippocampus and restore cognitive function in hyperammonemic rats. J. Neuroinflamm..

[11] Yates A.G., Pink R.C., Erdbrügger U., Siljander P.R., Dellar E.R., Pantazi P., Akbar N., Cooke W.R., Vatish M., Dias-Neto E. (2022). In sickness and in health: The functional role of extracellular vesicles in physiology and pathology in vivo. J. Extracell. Vesicles.

[12] Krämer-Albers E.M. (2022). Extracellular Vesicles at CNS barriers: Mode of action. Curr. Opin. Neurobiol..

[13] Yáñez-Mó M., Siljander P.R.-M., Andreu Z., Bedina Zavec A., Borràs F.E., Buzas E.I., Buzas K., Casal E., Cappello F., Carvalho J. (2015). Biological properties of extracellular vesicles and their physiological functions. J. Extracell. Vesicles.

[14] Raposo G., Stoorvogel W. (2013). Extracellular vesicles: Exosomes, microvesicles, and friends. J. Cell Biol..

[15] Colombo M., Raposo G., Théry C. (2014). Biogenesis, secretion, and intercellular interactions of exosomes and other extracellular vesicles. Annu. Rev. Cell Dev. Biol..

[16] Pfeiffer A., Petersen J.D., Falduto G.H., Anderson D.E., Zimmerberg J., Metcalfe D.D., Olivera A. (2022). Selective immunocapture reveals neoplastic human mast cells secrete distinct microvesicle- and exosome-like populations of KIT-containing extracellular vesicles. J. Extracell. Vesicles.

[17] Hallal S., Tűzesi Á., Grau G.E., Buckland M.E., Alexander K.L. (2022). Understanding the extracellular vesicle surface for clinical molecular biology. J. Extracell. Vesicles..

[18] Mathieu M., Martin-Jaular L., Lavieu G., Théry C. (2019). Specificities of secretion and uptake of exosomes and other extracellular vesicles for cell-to-cell communication. Nat. Cell Biol. Vol..

[19] Lischnig A., Bergqvist M., Ochiya T., Lässer C. (2022). Quantitative Proteomics Identifies Proteins Enriched in Large and Small Extracellular Vesicles. Mol. Cell. Proteom..

[20] Robbins P.D., Dorronsoro A., Booker C.N. (2016). Regulation of chronic inflammatory and immune processes by extracellular vesicles. Rev. J. Clin. Investig..

[21] Marar C., Starich B., Wirtz D. (2021). Extracellular vesicles in immunomodulation and tumor progression. Nat. Immunol..

[22] Schnatz A., Müller C., Brahmer A., Krämer-Albers E. (2021). Extracellular Vesicles in neural cell interaction and CNS homeostasis. Rev. FASEB Bioadv..

[23] Solana-Balaguer J., Campoy-Campos G., Martín-Flores N., Pérez-Sisqués L., Sitjà-Roqueta L., Kucukerden M., Gámez-Valero A., Coll-Manzano A., Martí E., Pérez-Navarro E. (2023). Neuron-derived extracellular vesicles contain synaptic proteins, promote spine formation, activate TrkB-mediated signalling and preserve neuronal complexity. J. Extracell. Vesicles.

[24] Gassama Y., Favereaux A. (2021). Emerging Roles of Extracellular Vesicles in the Central Nervous System: Physiology, Pathology, and Therapeutic Perspectives. Front. Cell. Neurosci..

[25] Visan K.S., Lobb R.J., Ham S., Lima L.G., Palma C., Edna C.P.Z., Wu L., Gowda H., Datta K.K., Hartel G. (2022). Comparative analysis of tangential flow filtration and ultracentrifugation, both combined with subsequent size exclusion chromatography, for the isolation of small extracellular vesicles. J. Extracell. Vesicles.

[26] Men Y., Yelick J., Jin S., Tian Y., Chiang M.S.R., Higashimori H., Brown E., Jarvis R., Yang Y. (2019). Exosome reporter mice reveal the involvement of exosomes in mediating neuron to astroglia communication in the CNS. Nat. Commun..

[27] Ma L., Wu Q., You Y., Zhang P., Tan D., Liang M., Huang Y., Gao Y., Ban Y., Chen Y. (2024). Neuronal small extracellular vesicles carrying miR-181c-5p contribute to the pathogenesis of epilepsy by regulating the protein kinase C-δ/glutamate transporter-1 axis in astrocytes. Glia.

[28] Bahrini I., Song J.-H., Diez D., Hanayama R. (2015). Neuronal exosomes facilitate synaptic pruning by up-regulating complement factors in microglia. Sci. Rep..

[29] Xian X., Cai L.-L., Li Y., Wang R.-C., Xu Y.-H., Chen Y.-J., Xie Y.-H., Zhu X.-L., Li Y.-F. (2022). Neuron secrete exosomes containing miR-9–5p to promote polarization of M1 microglia in depression. J. Nanobiotechnol..

[30] Wang H., Chen F.-S., Zhang Z.-L., Zhou H.-X., Ma H., Li X.-Q. (2021). MiR-126–3penriched extracellular vesicles from hypoxia-preconditioned VSC 4.1 neurons attenuate ischaemia-reperfusion-induced pain hypersensitivity by regulating the PIK3R2-mediated pathway. Mol. Neurobiol..

[31] Kaya Z., Belder N., Sever-Bahcekapili M., Donmez-Demir B., Erdener Ş.E., Bozbeyoglu N., Bagci C., Eren-Kocak E., Yemisci M., Karatas H. (2023). Vesicular HMGB1 release from neurons stressed with spreading depolarization enables confined inflammatory signaling to astrocytes. J. Neuroinflamm..

[32] Durur D.Y., Tastan B., Tufekci K.U., Olcum M., Uzuner H., Karakülah G., Yener G., Genc S. (2022). Alteration of miRNAs in Small Neuron-Derived Extracellular Vesicles of Alzheimer’s Disease Patients and the Effect of Extracellular Vesicles on Microglial Immune Responses. J. Mol. Neurosci..

[33] Fauré J., Lachenal G., Court M., Hirrlinger J., Chatellard-Causse C., Blot B., Grange J., Schoehn G., Goldberg Y., Boyer V. (2006). Exosomes are released by cultured cortical neurones. Mol. Cell. Neurosci..

[34] Yang Y., Boza-Serrano A., Dunning C.J.R., Clausen B.H., Lambertsen K.L., Deierborg T. (2018). Inflammation leads to distinct populations of extracellular vesicles from microglia. J. Neuroinflamm..

[35] Bianco F., Perrotta C., Novellino L., Francolini M., Riganti L., Menna E., Saglietti L., Schuchman E.H., Furlan R., Clementi E. (2009). Acid sphingomyelinase activity triggers microparticle release from glial cells. EMBO J..

[36] Antonucci F., Turola E., Riganti L., Caleo M., Gabrielli M., Perrotta C., Novellino L., Clementi E., Giussani P., Viani P. (2012). Microvesicles released from microglia stimulate synaptic activity via enhanced sphingolipid metabolism. EMBO J..

[37] Prada I., Gabrielli M., Turola E., Iorio A., D’arrigo G., Parolisi R., De Luca M., Pacifici M., Bastoni M., Lombardi M. (2018). Glia-to-neuron transfer of miRNAs via extracellular vesicles: A new mechanism underlying inflammation-induced synaptic alterations. Acta Neuropathol..

[38] Arvanitaki E.S., Goulielmaki E., Gkirtzimanaki K., Niotis G., Tsakani E., Nenedaki E., Rouska I., Kefalogianni M., Xydias D., Kalafatakis I. (2024). Microglia-derived extracellular vesicles trigger age-related neurodegeneration upon DNA damage. Proc. Natl. Acad. Sci. USA.

[39] Gao S., Bai L., Jia S., Meng C. (2022). Small Extracellular Vesicles of M1-BV2 Microglia Induce Neuronal PC12 Cells Apoptosis via the Competing Endogenous Mechanism of CircRNAs. Genes.

[40] Qi Z., Yu Y., Su Y., Cao B., Shao H., Yang J.-J. (2023). M1-Type Microglia-Derived Extracellular Vesicles Overexpressing IL-1R1 Promote Postoperative Cognitive Dysfunction by Regulating Neuronal Inflammation. Inflammation.

[41] Zhou Z., Li C., Bao T., Zhao X., Xiong W., Luo C., Yin G., Fan J. (2022). Exosome-Shuttled miR-672–5p from Anti-Inflammatory Microglia Repair Traumatic Spinal Cord Injury by Inhibiting AIM2/ASC/Caspase-1 Signaling Pathway Mediated Neuronal Pyroptosis. J. Neurotrauma.

[42] Gabrielli M., Prada I., Joshi P., Falcicchia C., D’arrigo G., Rutigliano G., Battocchio E., Zenatelli R., Tozzi F., Radeghieri A. (2023). Microglial large extracellular vesicles propagate early synaptic dysfunction in Alzheimer’s disease. Brain.

[43] Fan C., Li Y., Lan T., Wang W., Long Y., Yu S.Y. (2021). Microglia secrete miR-146a-5p-containing exosomes to regulate neurogenesis in depression. Mol. Ther..

[44] Ji X.-Y., Guo Y.-X., Wang L.-B., Wu W.-C., Wang J.-Q., He J., Gao R., Rasouli J., Gao M.-Y., Wang Z.-H. (2024). Microglia-derived exosomes modulate myelin regeneration via miR-615–5p/MYRF axis. J. Neuroinflamm..

[45] Drago F., Lombardi M., Prada I., Gabrielli M., Joshi P., Cojoc D., Franck J., Fournier I., Vizioli J., Verderio C. (2017). ATP modifies the proteome of extracellular vesicles released by microglia and influences their action on astrocytes. Front. Pharmacol..

[46] Taylor A.R., Robinson M.B., Gifondorwa D.J., Tytell M., Milligan C.E. (2007). Regulation of heat shock protein 70 release in astrocytes: Role of signaling kinases. Dev. Neurobiol..

[47] Hajrasouliha A.R., Jiang G., Lu Q., Lu H., Kaplan H.J., Zhang H.-G., Shao H. (2013). Exosomes from retinal astrocytes contain anti-angiogenic components that inhibit laser-induced choroidal neovascularization. J. Biol. Chem..

[48] Basso M., Pozzi S., Tortarolo M., Fiordaliso F., Bisighini C., Pasetto L., Spaltro G., Lidonnici D., Gensano F., Battaglia E. (2013). Mutant copperzinc superoxide dismutase (SOD1) induces protein secretion pathway alterations and exosome release in astrocytes: Implications for disease spreading and motor neuron pathology in amyotrophic lateral sclerosis. J. Biol. Chem..

[49] Hu G., Yao H., Chaudhuri A.D., Duan M., Yelamanchili S.V., Wen H., Cheney P.D., Fox H.S., Buch S. (2012). Exosome-mediated shuttling of microRNA-29 regulates HIV Tat and morphine-mediated neuronal dysfunction. Cell Death Dis..

[50] Takenouchi T., Tsukimoto M., Iwamaru Y., Sugama S., Sekiyama K., Sato M., Kojima S., Hashimoto M., Kitani H. (2015). Extracellular ATP induces unconventional release of glyceraldehyde-3-phosphate dehydrogenase from microglial cells. Immunol. Lett..

[51] La Torre M.E., Panaro M.A., Ruggiero M., Polito R., Cianciulli A., Filannino F.M., Lofrumento D.D., Antonucci L., Benameur T., Monda V. (2022). Extracellular Vesicles Cargo in Modulating Microglia Functional Responses. Biology.

[52] Wang S., Cesca F., Loers G., Schweizer M., Buck F., Benfenati F., Schachner M., Kleene R. (2011). Synapsin I is an oligomannose-carrying glycoprotein, acts as an oligomannosebinding lectin, and promotes neurite outgrowth and neuronal survival when released via glia-derived exosomes. J. Neurosci..

[53] Wang G., Dinkins M., He Q., Zhu G., Poirier C., Campbell A., Mayer-Proschel M., Bieberich E. (2012). Astrocytes secrete exosomes enriched with proapoptotic ceramide and prostate apoptosis response 4 (PAR4): Potential mechanism of apoptosis induction in Alzheimer disease (AD). J. Biol. Chem..

[54] Zhu Z., Quadri Z., Crivelli S.M., Elsherbini A., Zhang L., Tripathi P., Qin H., Roush E., Spassieva S.D., Nikolova-Karakashian M. (2022). Neutral Sphingomyelinase 2 Mediates Oxidative Stress Effects on Astrocyte Senescence and Synaptic Plasticity Transcripts. Mol. Neurobiol..

[55] Tonoli E., Verduci I., Gabrielli M., Prada I., Forcaia G., Coveney C., Savoca M.P., Boocock D.J., Sancini G., Mazzanti M. (2022). Extracellular transglutaminase-2, nude or associated with astrocytic extracellular vesicles, modulates neuronal calcium homeostasis. Prog. Neurobiol..

[56] Sun H., Cao X., Gong A., Huang Y., Xu Y., Zhang J., Sun J., Lv B., Li Z., Guan S. (2022). Extracellular vesicles derived from astrocytes facilitated neurite elongation by activating the Hippo pathway. Exp. Cell Res..

[57] Krämer-Albers E.-M., Bretz N., Tenzer S., Winterstein C., Möbius W., Berger H., Nave K.-A., Schild H., Trotter J. (2007). Oligodendrocytes secrete exosomes containing major myelin and stress-protective proteins: Trophic support for axons?. Proteom. Clin. App..

[58] Fitzner D., Schnaars M., van Rossum D., Krishnamoorthy G., Dibaj P., Bakhti M., Regen T., Hanisch U.-K., Simons M. (2011). Selective transfer of exosomes from oligodendrocytes to microglia by macropinocytosis. J. Cell Sci..

[59] Frühbeis C., Fröhlich D., Kuo W.P., Amphornrat J., Thilemann S., Saab A.S., Kirchhoff F., Möbius W., Goebbels S., Nave K.-A. (2013). Neurotransmitter-triggered transfer of exosomes mediates oligodendrocyte-neuron communication. PLoS Biol..

[60] Zhang H., Xie X., Xu S., Wang C., Sun S., Song X., Li R., Li N., Feng Y., Duan H. (2024). Oligodendrocyte-derived exosomes-containing SIRT2 ameliorates depressive-like behaviors and restores hippocampal neurogenesis and synaptic plasticity via the AKT/GSK-3β pathway in depressed mice. CNS Neurosci. Ther..

[61] Yuan D., Zhao Y., Banks W.A., Bullock K.M., Haney M., Batrakova E., Kabanov A.V. (2017). Macrophage exosomes as natural nanocarriers for protein delivery to inflamed brain. Biomaterials.

[62] SáEnz-Cuesta M., Osorio-Querejeta I., Otaegui D. (2014). Extracellular Vesicles in Multiple Sclerosis: What Are They Telling Us?. Rev. Front. Cell. Neurosci..

[63] Sáenz-Cuesta M., Irizar H., Castillo-Triviño T., Muñoz-Culla M., Osorio-Querejeta I., Prada A., Sepúlveda L., López-Mato M.P., de Munain A.L., Comabella M. (2014). Circulating microparticles reflect treatment effects and clinical status in multiple sclerosis. Biomark. Med..

[64] Matsumoto J., Stewart T., Sheng L., Li N., Bullock K., Song N., Shi M., Banks W.A., Zhang J. (2017). Transmission of alpha-synuclein containing erythrocyte-derived extracellular vesicles across the blood-brain barrier via adsorptive mediated transcytosis: Another mechanism for initiation and progression of Parkinson’s disease?. Acta Neuropathol. Commun..

[65] Ridder K., Keller S., Dams M., Rupp A.K., Schlaudraff J., Del Turco D., Starmann J., Macas J., Karpova D., Devraj K. (2018). Extracellular vesicle-mediated transfer of genetic information between the hematopoietic system and the brain in response to inflammation. PLoS Biol..

[66] Schindler C.R., Hörauf J.A., Weber B., Schaible I., Marzi I., Henrich D., Leppik L. (2024). Identification of novel blood-based extracellular vesicles biomarker candidates with potential specificity for traumatic brain injury in polytrauma patients. Front. Immunol..

[67] Ricklefs F.L., Wollmann K., Salviano-Silva A., Drexler R., Maire C.L., Kaul M.G., Reimer R., Schüller U., Heinemann S., Kolbe K. (2024). Circulating extracellular vesicles as biomarker for diagnosis, prognosis and monitoring in glioblastoma patients. Neuro Oncol..

[68] Blommer J., Pitcher T., Mustapic M., Eren E., Yao P.J., Vreones M.P., Pucha K.A., Dalrymple-Alford J., Shoorangiz R., Meissner W.G. (2022). Extracellular vesicle biomarkers for cognitive impairment in Parkinson’s disease. Brain.

[69] Mustapic M., Eitan E., Werner J.K., Berkowitz S.T., Lazaropoulos M.P., Tran J., Goetzl E.J., Kapogiannis D. (2017). Plasma Extracellular Vesicles Enriched for Neuronal Origin: A Potential Window into Brain Pathologic Processes. Front. Neurosci..

[70] Huo S., Kränkel N., Nave A.H., Sperber P.S., Rohmann J.L., Piper S.K., Heuschmann P.U., Landmesser U., Endres M., Siegerink B. (2021). Endothelial and leukocyte-derived microvesicles and cardiovascular risk after stroke: PROSCIS-B. Neurology.

[71] Hisada Y., Mackman N. (2019). Measurement of tissue factor activity in extracellular vesicles from human plasma samples. Res. Pract. Thromb. Haemost..

[72] Qu M., Lin Q., Huang L., Fu Y., Wang L., He S., Fu Y., Yang S., Zhang Z., Zhang L. (2018). Dopamine-loaded blood exosomes targeted to brain for better treatment of Parkinson’s disease. J. Contr. Release.

[73] Zhang M., Gong W., Zhang D., Ji M., Chen B., Chen B., Li X., Zhou Y., Dong C., Wen G. (2022). Ageing related thyroid deficiency increases brain-targeted transport of liver-derived ApoE4-laden exosomes leading to cognitive impairment. Cell Death Dis..

[74] Li J.J., Wang B., Kodali M.C., Chen C., Kim E., Patters B.J., Lan L., Kumar S., Wang X., Yue J. (2018). In vivo evidence for the contribution of peripheral circulating inflammatory exosomes to neuroinflammation. J. Neuroinflamm..

[75] Zhang L., Liu H., Jia L., Lyu J., Sun Y., Yu H., Li H., Liu W., Weng Y., Yu W. (2019). Exosomes mediate hippocampal and cortical neuronal injury induced by hepatic ischemia-reperfusion injury through activating pyroptosis in rats. Oxid. Med. Cell. Longev..

[76] Qi Z., Zhao Y., Su Y., Cao B., Yang J.-J., Xing Q. (2021). Serum extracellular vesicle-derived miR-124–3p as a diagnostic and predictive marker for early-stage acute ischemic stroke. Front. Mol. Biosci..

[77] Morad G., Carman C.V., Hagedorn E.J., Perlin J.R., Zon L.I., Mustafaoglu N., Park T.E., Ingber D.E., Daisy C.C., Moses M.A. (2019). Tumor-derived extracellular vesicles breach the intact blood-brain barrier via transcytosis. ACS Nano.

[78] Kuroda H., Tachikawa M., Yagi Y., Umetsu M., Nurdin A., Miyauchi E., Watanabe M., Uchida Y., Terasaki T. (2019). Cluster of differentiation 46 is the major receptor in human blood-brain barrier endothelial cells for uptake of exosomes derived from brain-metastatic melanoma cells (SK-Mel-28). Mol. Pharm..

[79] Balusu S., Van Wonterghem E., De Rycke R., Raemdonck K., Stremersch S., Gevaert K., Brkic M., Demeestere D., Vanhooren V., Hendrix A. (2016). Identification of a novel mechanism of blood-brain communication during peripheral inflammation via choroid plexus-derived extracellular vesicles. EMBO Mol. Med..

[80] Verderio C., Muzio L., Turola E., Bergami A., Novellino L., Ruffini F., Riganti L., Corradini I., Francolini M., Garzetti L. (2012). Myeloid microvesicles are a marker and therapeutic target for neuroinflammation. Ann. Neurol..

[81] Vandendriessche C., Balusu S., Van Cauwenberghe C., Brkic M., Pauwels M., Plehiers N., Bruggeman A., Dujardin P., Van Imschoot G., Van Wonterghem E. (2021). Importance of extracellular vesicle secretion at the blood-cerebrospinal fluid interface in the pathogenesis of Alzheimer’s disease. Acta Neuropathol. Commun..

[82] Wang J., Li L., Zhang Z., Zhang X., Zhu Y., Zhang C., Bi Y. (2022). Extracellular vesicles mediate the communication of adipose tissue with brain and promote cognitive impairment associated with insulin resistance. Cell Metab..

[83] Kojima M., Costantini T.W., Eliceiri B.P., Chan T.W., Baird A., Coimbra R. (2018). Gut epithelial cell-derived exosomes trigger posttrauma immune dysfunction. J. Trauma. Acute Care Surg..

[84] Joshi B.S., Zuhorn I.S. (2021). Heparan sulfate proteoglycan-mediated dynamin-dependent transport of neural stem cell exosomes in an in vitro blood-brain barrier model. Eur. J. Neurosci..

[85] Wei X., Liu C., Wang H., Wang L., Xiao F., Guo Z., Zhang H. (2016). Surface phosphatidylserine is responsible for the internalization on microvesicles derived from hypoxia-induced human bone marrow mesenchymal stem cells into human endothelial cells. PLoS ONE.

[86] Otero-Ortega L., Laso-Garcia F., Gomez-de F.M., Rodriguez-Frutos B., Pascual-Guerra J., Fuentes B., Diez-Tejedor E., Gutierrez-Fernandez M. (2017). White matter repair after extracellular vesicles administration in an experimental animal model of subcortical stroke. Sci. Rep..

[87] Jiang M., Wang H., Jin M., Yang X., Ji H., Jiang Y., Zhang H., Wu F., Wu G., Lai X. (2018). Exosomes from MiR-30d-5p-ADSCs reverse acute ischemic stroke-induced, autophagy mediated brain injury by promoting M2 microglial/macrophage polarization. Cell. Physiol. Biochem..

[88] Lv H., Li J., Che Y. (2021). miR-31 from adipose stem cell-derived extracellular vesicles promotes recovery of neurological function after ischemic stroke by inhibiting TRAF6 and IRF5. Exp. Neurol..

[89] Feng N., Jia Y., Huang X. (2019). Exosomes from adipose-derived stem cells alleviate neural injury caused by microglia activation via suppressing NF-kB and MAPK pathway. J. Neuroimmunol..

[90] Katsuda T., Tsuchiya R., Kosaka N., Yoshioka Y., Takagaki K., Oki K., Takeshita F., Sakai Y., Kuroda M., Ochiya T. (2013). Human adipose tissue-derived mesenchymal stem cells secrete functional neprilysin-bound exosomes. Sci. Rep..

[91] Geng W., Tang H., Luo S., Lv Y., Liang D., Kang X., Hong W. (2019). Exosomes from miRNA-126-modified ADSCs promotes functional recovery after stroke in rats by improving neurogenesis and suppressing microglia activation. Am. J. Transl. Res..

[92] Lee M., Ban J.J., Kim K.Y., Jeon G.S., Im W., Sung J.J., Kim M. (2016). Adipose-derived stem cell exosomes alleviate pathology of amyotrophiclateral sclerosis in vitro. Biochem. Biophys. Res. Commun..

[93] Yang Y., Cai Y., Zhang Y., Liu J., Xu Z. (2018). Exosomes secreted by adipose-derived stem cells contribute to angiogenesis of brain microvascular endothelial cells following oxygen-glucose deprivation in vitro through MicroRNA-181b/TRPM7 axis. J. Mol. Neurosci..

[94] Yang H., Tu Z., Yang D., Hu M., Zhou L., Li Q., Yu B., Hou S. (2022). Exosomes from hypoxic pre-treated ADSCs attenuate acute ischemic stroke-induced brain injury via delivery of circ-Rps5 and promote M2 microglia/macrophage polarization. Neurosci. Lett..

[95] Wei S., Peng W., Mai Y., Li K., Wei W., Hu L., Zhu S., Zhou H., Jie W., Wei Z. (2020). Outer membrane vesicles enhance tau phosphorylation and contribute to cognitive impairment. J. Cell Physiol..

[96] Lee K.E., Kim J.K., Han S.K., Lee D.Y., Lee H.J., Yim S.V., Kim D.H. (2020). The extracellular vesicle of gut microbial *Paenalcaligenes hominis* is a risk factor for vagus nerve-mediated cognitive impairment. Microbiome.

[97] Zakharzhevskaya N.B., Vanyushkina A.A., Altukhov I.A., Shavarda A.L., Butenko I.O., Rakitina D.V., Nikitina A.S., Manolov A.I., Egorova A.N., Kulikov E.E. (2017). Outer membrane vesicles secreted by pathogenic and nonpathogenic Bacteroides fragilis represent different metabolic activities. Sci. Rep..

[98] Choi J., Kim Y.K., Han P.L. (2019). Extracellular vesicles derived from Lactobacillus plantarum increase BDNF expression in cultured hippocampal neurons and produce antidepressant-like effects in mice. Exp. Neurobiol..

[99] Choi J., Kwon H., Kim Y.K., Han P.L. (2022). Extracellular vesicles from gram-positive and gram-negative probiotics remediate stress-induced depressive behavior in mice. Mol. Neurobiol..

[100] Ha J.Y., Choi S.Y., Lee J.H., Hong S.H., Lee H.J. (2020). Delivery of periodontopathogenic extracellular vesicles to brain monocytes and microglial IL-6 promotion by RNA cargo. Front. Mol. Biosci..

[101] Han E.C., Choi S.Y., Lee Y., Park J.W., Hong S.H., Lee H.J. (2019). Extracellular RNAs in periodontopathogenic outer membrane vesicles promote TNF-alpha production in human macrophages and cross the blood-brain barrier in mice. FASEB J..

[102] Inotsuka R., Uchimura K., Yamatsu A., Kim M., Katakura Y. (2020). Gamma-aminobutyric acid (GABA) activates neuronal cells by inducing the secretion of exosomes from intestinal cells. Food Funct..

[103] Larabi A., Dalmasso G., Delmas J., Barnich N., Nguyen H. (2020). Exosomes transfer miRNAs from cell-to-cell to inhibit autophagy during infection with Crohn’s disease-associated adherent-invasive *E. coli*. Gut Microbes.

[104] Xi S., Wang Y., Wu C., Peng W., Zhu Y., Hu W. (2021). Intestinal epithelial cell exosome launches IL-1beta-mediated neuron injury in sepsis-associated encephalopathy. Front. Cell. Infect. Microbiol..

[105] Ayyar K.K., Moss A.C. (2021). Exosomes in intestinal inflammation. Front. Pharmacol..

[106] Budnik V., Ruiz-Cañada C., Wendler F. (2016). Extracellular vesicles round off communication in the nervous system. Nat. Rev. Neurosci..

[107] Li T., Tan X., Li S., Al-Nusaif M., Le W. (2021). Role of Glia-Derived Extracellular Vesicles in Neurodegenerative Diseases. Front. Aging Neurosci..

[108] Peferoen L., Kipp M., van der Valk P., van Noort J.M., Amor S. (2014). Oligodendrocyte–microglia cross-talk in the central nervous system. Immunology.

[109] Paschon V., Takada S.H., Ikebara J.M., Sousa E., Raeisossadati R., Ulrich H., Kihara A.H. (2015). Interplay between exosomes, microRNAs and Toll-like receptors in brain disorders. Mol. Neurobiol..

[110] Gupta A., Pulliam L. (2014). Exosomes as mediators of neuroinflammation. J. Neuroinflamm..

[111] Pascual M., Ibáñez F., Guerri C. (2020). Exosomes as mediators of neuron-glia communication in neuroinflammation. Neural Regen. Res..

[112] Taoro-González L., Cabrera-Pastor A., Sancho-Alonso M., Arenas Y.M., Meseguer-Estornell F., Balzano T., ElMlili N., Felipo V. (2019). Differential role of interleukin-1β in neuroinflammation-induced impairment of spatial and nonspatial memory in hyperammonemic rats. FASEB J..

[113] Taoro-Gonzalez L., Arenas Y.M., Cabrera-Pastor A., Felipo V. (2018). Hyperammonemia alters membrane expression of GluA1 and GluA2 subunits of AMPA receptors in hippocampus by enhancing activation of the IL-1 receptor: Underlying mechanisms. J. Neuroinflamm..

[114] Arenas Y.M., Cabrera-Pastor A., Juciute N., Mora-Navarro E., Felipo V. (2020). Blocking glycine receptors reduces neuroinflammation and restores neurotransmission in cerebellum through ADAM17-TNFR1-NF-κβ pathway. J. Neuroinflamm..

[115] Cabrera-Pastor A., Arenas Y.M., Taoro-Gonzalez L., Montoliu C., Felipo V. (2019). Chronic hyperammonemia alters extracellular glutamate, glutamine and GABA and membrane expression of their transporters in rat cerebellum. Modulation by extracellular cGMP. Neuropharmacology.

[116] Frühbeis C., Fröhlich D., Kuo W.P., Krämer-Albers E.M. (2013). Extracellular vesicles as mediators of neuron-glia communication. Front. Cell. Neurosci..

[117] Turola E., Furlan R., Bianco F., Matteoli M., Verderio C. (2012). Microglial microvesicle secretion and intercellular signaling. Front. Physiol..

[118] Pan J.-J., Qi L., Wang L., Liu C., Song Y., Mamtilahun M., Hu X., Li Y., Chen X., Khan H. (2024). M2 Microglial Extracellular Vesicles Attenuated Blood-brain Barrier Disruption via MiR-23a-5p in Cerebral Ischemic Mice. Aging Dis..

[119] Kim S.J., Russell A.E., Wang W., Gemoets D.E., Sarkar S.N., Simpkins J.W., Brown C.M. (2022). miR-146a Dysregulates Energy Metabolism During Neuroinflammation. J. Neuroimmune Pharmacol..

[120] Chaudhuri A.D., Dasgheyb R.M., DeVine L.R., Bi H., Cole R.N., Haughey N.J. (2020). Stimulus-dependent modifications in astrocyte-derived extracellular vesicle cargo regulate neuronal excitability. Glia.

[121] D’Arrigo G., Gabrielli M., Scaroni F., Swuec P., Amin L., Pegoraro A., Adinolfi E., Di Virgilio F., Cojoc D., Legname G. (2021). Astrocytes-derived extracellular vesicles in motion at the neuron surface: Involvement of the prion protein. J. Extracell. Vesicles.

[122] Frühbeis C., Kuo-Elsner W.P., Müller C., Barth K., Peris L., Tenzer S., Möbius W., Werner H.B., Nave K.-A., Fröhlich D. (2020). Oligodendrocytes support axonal transport and maintenance via exosome secretion. PLoS Biol..

[123] Saquel C., Catalan R.J., Lopez-Leal R., Ramirez R.A., Necuñir D., Wyneken U., Lamaze C., Court F.A. (2022). Neuronal activity-dependent ATP enhances the pro-growth effect of repair Schwann cell extracellular vesicles by increasing their miRNA-21 loading. Front. Cell. Neurosci..

[124] Perry V.H., Teeling J. (2013). Microglia and macrophages of the central nervous system: The contribution of microglia priming and systemic inflammation to chronic neurodegeneration. Semin. Immunopathol..

[125] Langen U.H., Ayloo S., Gu C. (2019). Development and cell biology of the blood-brain barrier. Annu. Rev. Cell Dev. Biol..

[126] Cousins O., Hodges A., Schubert J., Veronese M., Turkheimer F., Miyan J., Engelhardt B., Roncaroli F. (2022). The blood-CSF-brain route of neurological disease: The indirect pathway into the brain. Rev. Neuropathol. Appl. Neurobiol..

[127] Verweij F.J., Balaj L., Boulanger C.M., Carter D.R.F., Compeer E.B., D’Angelo G., El Andaloussi S., Goetz J.G., Gross J.C., Hyenne V. (2021). The power of imaging to understand extracellular vesicle biology in vivo. Nat. Methods.

[128] Venkat P., Chen J., Chopp M. (2018). Exosome-mediated amplification of endogenous brain repair mechanisms and brain and systemic organ interaction in modulating neurological outcome after stroke. J. Cereb. Blood Flow Metab..

[129] Doehner W., Ural D., Haeusler K.G., Čelutkienė J., Bestetti R., Cavusoglu Y., Peña-Duque M.A., Glavas D., Iacoviello M., Laufs U. (2018). Heart and brain interaction in patients with heart failure: Overview and proposal for a taxonomy. A position paper from the Study Group on Heart and Brain Interaction of the Heart Failure Association. Eur. J. Heart Fail..

[130] Yang Y., Rosenberg G.A. (2011). Blood-brain barrier breakdown in acute and chronic cerebrovascular disease. Stroke.

[131] Edwardson M.A., Mitsuhashi M., Van Epps D. (2024). Elevation of astrocyte-derived extracellular vesicles over the first month post-stroke in humans. Sci. Rep..

[132] Chen Z., Venkat P., Seyfried D., Chopp M., Yan T., Chen J. (2017). Brain-heart interaction: Cardiac complications after stroke. Circ. Res..

[133] Liu M.-L., Williams K.J. (2012). Microvesicles: Potential markers and mediators of endothelial dysfunction. Curr. Opin. Endocrinol. Diabetes Obes..

[134] Zheng X., Hermann D.M., Bähr M., Doeppner T.R. (2021). The role of small extracellular vesicles in cerebral and myocardial ischemia—Molecular signals, treatment targets, and future clinical translation. Stem Cells.

[135] Long G., Wang F., Li H., Yin Z., Sandip C., Lou Y., Wang Y., Chen C., Wang D.W. (2013). Circulating miR-30a, miR-126 and let-7b as biomarker for ischemic stroke in humans. BMC Neurol..

[136] Chen J., Cui C., Yang X., Xu J., Venkat P., Zacharek A., Yu P., Chopp M. (2017). MiR-126 affects brain-heart interaction after cerebral ischemic stroke. Transl. Stroke Res..

[137] Wei X.J., Han M., Yang F.Y., Wei G.C., Liang Z.G., Yao H., Ji C.W., Xie R.S., Gong C.L., Tian Y. (2015). Biological significance of miR-126 expression in atrial fibrillation and heart failure. Braz. J. Med. Biol. Res..

[138] Zeng L., He X., Wang Y., Tang Y., Zheng C., Cai H., Liu J., Wang Y., Fu Y., Yang G.-Y. (2014). MicroRNA-210 overexpression induces angiogenesis and neurogenesis in the normal adult mouse brain. Gene Ther..

[139] Fan Z.-G., Qu X.-L., Chu P., Gao Y.-L., Gao X.-F., Chen S.-L., Tian N.-L. (2018). MicroRNA-210 promotes angiogenesis in acute myocardial infarction. Mol. Med. Rep..

[140] Shang J., Deguchi K., Ohta Y., Liu N., Zhang X., Tian F., Yamashita T., Ikeda Y., Matsuura T., Funakoshi H. (2011). Strong neurogenesis, angiogenesis, synaptogenesis, and antifibrosis of hepatocyte growth factor in rats brain after transient middle cerebral artery occlusion. J. Neurosci. Res..

[141] Zhang Y., Zhang Y., Chopp M., Pang H., Zhang Z.G., Mahmood A., Xiong Y. (2021). MiR-17–92 cluster-enriched exosomes derived from human bone marrow mesenchymal stromal cells improve tissue and functional recovery in rats after traumatic brain injury. J. Neurotrauma.

[142] Chen J., Huang Z.-P., Seok H.Y., Ding J., Kataoka M., Zhang Z., Hu X., Wang G., Lin Z., Wang S. (2013). mir-17–92 cluster is required for and sufficient to induce cardiomyocyte proliferation in postnatal and adult hearts. Circ. Res..

[143] Liu X.S., Chopp M., Wang X.L., Zhang L., Hozeska-Solgot A., Tang T., Kassis H., Zhang R.L., Chen C., Xu J. (2013). MicroRNA-17–92 cluster mediates the proliferation and survival of neural progenitor cells after stroke. J. Biol. Chem..

[144] Liu Y., Zhang J., Han R., Liu H., Sun D., Liu X. (2015). Downregulation of serum brain specific microRNA is associated with inflammation and infarct volume in acute ischemic stroke. J. Clin. Neurosci..

[145] He F., Liu H., Guo J., Yang D., Yu Y., Yu J., Yan X., Hu J., Du Z. (2018). Inhibition of microRNA-124 reduces cardiomyocyte apoptosis following myocardial infarction via targeting STAT3. Cell Physiol. Biochem. Int. Biochem..

[146] Zhao R.-T., Zhou J., Dong X.-L., Bi C.-W., Jiang R.-C., Dong J.-F., Tian Y., Yuan H.-J., Zhang J.-N. (2018). Circular ribonucleic acid expression alteration in exosomes from the brain extracellular space after traumatic brain injury in mice. J. Neurotrauma.

[147] Hajer G.R., van Haeften T.W., Visseren F.L. (2008). Adipose tissue dysfunction in obesity, diabetes, and vascular diseases. Eur. Heart J..

[148] Thomou T., Mori M.A., Dreyfuss J.M., Konishi M., Sakaguchi M., Wolfrum C., Rao T.N., Winnay J.N., Garcia-Martin R., Grinspoon S.K. (2017). Adipose-derived circulating miRNAs regulate gene expression in other tissues. Nature.

[149] Felipo V. (2013). Hepatic encephalopathy: Effects of liver failure on brain function. Nat. Rev. Neurosci..

[150] Cabrera-Pastor A., Balzano T., Hernández-Rabaza V., Malaguarnera M., Llansola M., Felipo V. (2018). Increasing extracellular cGMP in cerebellum in vivo reduces neuroinflammation, GABAergic tone and motor in-coordination in hyperammonemic rats. Brain Behav. Immun..

[151] Cabrera-Pastor A., Hernandez-Rabaza V., Taoro-Gonzalez L., Balzano T., Llansola M., Felipo V. (2016). In vivo administration of extracellular cGMP normalizes TNF-α and membrane expression of AMPA receptors in hippocampus and spatial reference memory but not IL-1β, NMDA receptors in membrane and working memory in hyperammonemic rats. Brain Behav. Immun..

[152] Cabrera-Pastor A., Taoro-González L., López-Merino E., Celma F., Llansola M., Felipo V. (2018). Chronic hyperammonemia alters in opposite ways membrane expression of GluA1 and GluA2 AMPA receptor subunits in cerebellum. Molecular mechanisms involved. Biochim Biophys Acta Mol Basis Dis..

[153] Malaguarnera M., Balzano T., Castro M.C., Llansola M., Felipo V. (2021). The Dual Role of the GABAA Receptor in Peripheral Inflammation and Neuroinflammation: A Study in Hyperammonemic Rats. Int. J. Mol. Sci..

[154] Malaguarnera M., Llansola M., Balzano T., Gómez-Giménez B., Antúnez-Muñoz C., Martínez-Alarcón N., Mahdinia R., Felipo V. (2019). Bicuculline Reduces Neuroinflammation in Hippocampus and Improves Spatial Learning and Anxiety in Hyperammonemic Rats. Role of Glutamate Receptors. Front. Pharmacol..

[155] Socala K., Doboszewska U., Szopa A., Serefko A., Wlodarczyk M., Zielinska A., Poleszak E., Fichna J., Wlaz P. (2021). The role of microbiota-gut-brain axis in neuropsychiatric and neurological disorders. Pharmacol. Res..

[156] Zhao L., Ye Y., Gu L., Jian Z., Stary C.M., Xiong X. (2021). Extracellular vesicle-derived miRNA as a novel regulatory system for bi-directional communication in gut-brain-microbiota axis. J. Transl. Med..

[157] Macia L., Nanan R., Hosseini-Beheshti E., Grau G.E. (2019). Host- and microbiota-derived extracellular vesicles, immune function, and disease development. Int. J. Mol. Sci..

[158] Pirolli N.H., Bentley W.E., Jay S.M. (2021). Bacterial extracellular vesicles and the gut-microbiota brain axis: Emerging roles in communication and potential as therapeutics. Adv. Biol..

[159] Abdel-Haq R., Schlachetzki J., Glass C.K., Mazmanian S.K. (2019). Microbiome-microglia connections via the gut-brain axis. J. Exp. Med..

